# Proteomic analysis of human mesenchymal stromal cell secretomes: a systematic comparison of the angiogenic potential

**DOI:** 10.1038/s41536-019-0070-y

**Published:** 2019-04-16

**Authors:** Debora Kehl, Melanie Generali, Anna Mallone, Manfred Heller, Anne-Christine Uldry, Phil Cheng, Benjamin Gantenbein, Simon P. Hoerstrup, Benedikt Weber

**Affiliations:** 1 Institute for Regenerative Medicine (IREM), University of Zurich, Zurich, Switzerland; 2 Mass Spectrometry and Proteomics Core Facility, University of Bern, Bern, Switzerland; 3 Department for BioMedical Research (DBMR), University of Bern, Bern, Switzerland; 4 Department of Dermatology, University Hospital Zurich, Zurich, Switzerland; 5 Center for Applied Biotechnology and Molecular Medicine (CABMM), University of Zurich, Zurich, Switzerland; 6 Tissue and Organ Mechanobiology, Institute for Surgical Technology and Biomechanics, University of Bern, Bern, Switzerland; 7 Department for Orthopaedics and Traumatology, Insel University Hospital, University of Bern, Bern, Switzerland; 8 Zurich Center for Integrative Human Physiology (ZHIP), University of Zurich, Zurich, Switzerland; 9 Wyss Zurich, University of Zurich and ETH Zurich, Zurich, Switzerland; 10 Skin and Endothelium Research Division (SERD), Department of Dermatology, Medical University of Vienna, Vienna, Austria

## Abstract

Human mesenchymal stromal cell (hMSC) secretomes have shown to influence the microenvironment upon injury, promoting cytoprotection, angiogenesis, and tissue repair. The angiogenic potential is of particular interest for the treatment of ischemic diseases. Interestingly, hMSC secretomes isolated from different tissue sources have shown dissimilarities with respect to their angiogenic profile. This study compares angiogenesis of hMSC secretomes from adipose tissue (hADSCs), bone marrow (hBMSCs), and umbilical cord Wharton’s jelly (hWJSCs). hMSC secretomes were obtained under xenofree conditions and analyzed by liquid chromatography tandem mass spectrometry (LC/MS-MS). Biological processes related to angiogenesis were found to be enriched in the proteomic profile of hMSC secretomes. hWJSC secretomes revealed a more complete angiogenic network with higher concentrations of angiogenesis related proteins, followed by hBMSC secretomes. hADSC secretomes lacked central angiogenic proteins and expressed most detected proteins to a significantly lower level. In vivo all secretomes induced vascularization of subcutaneously implanted Matrigel plugs in mice. Differences in secretome composition were functionally analyzed with monocyte and endothelial cell (EC) in vitro co-culture experiments using vi-SNE based multidimensional flow cytometry data analysis. Functional responses between hBMSC and hWJSC secretomes were comparable, with significantly higher migration of CD14^++^ CD16^−^ monocytes and enhanced macrophage differentiation compared with hADSC secretomes. Both secretomes also induced a more profound pro-angiogenic phenotype of ECs. These results suggest hWJSCs secretome as the most potent hMSC source for inflammation-mediated angiogenesis induction, while the potency of hADSC secretomes was lowest. This systematic analysis may have implication on the selection of hMSCs for future clinical studies.

## Introduction

Mesenchymal stromal cells (MSCs) are a heterogeneous population of non-clonal cells containing a multipotent stem cell fraction. Since their introduction for the use in regenerative medicine, the number of pre-clinical and first clinical studies has continuously increased over the last decades.^1^ More than 850 registered human clinicals trials have been performed by September 2018 (ClinicalTrial.gov) and these numbers are expected to further increase in the near future. Initial MSC research focused on the multilineage differentiation of cells towards desired phenotypes. However, it has become evident that functional benefits exerted by MSCs upon transplantation are rather due to the release of paracrine factors and biologically relevant molecules to the neighboring diseased or injured tissue.^2,3^ The MSC secretome influences the microenvironment upon injury, promoting cytoprotection, and tissue repair of the damaged area. These effects are of particular interest for the treatment of ischemically damaged tissues in which promoting vascularization and reinstalling perfusion via angiogenesis is crucial for increasing potential tissue rescue and thereby preventing fibrosis.

Angiogenic factors released by MSCs include among others basic fibroblast growth factor (bFGF), vascular endothelial growth factor (VEGF), transforming growth factor beta (TGF-β), platelet-derived growth factor (PDGF), angiopoietin-1 (ANG-1), placental growth factor (PIGF), interleukin 6 (IL-6), and monocyte chemoattractant protein 1 (MCP-1).^4^ The MSC secretome positively stimulates angiogenesis in vitro and in vivo^4^ and the angiogenic activity can be significantly inhibited by neutralizing antibodies against specific cytokines, such as VEGF, MCP-1, and IL-6.^5^ In addition, exosomes mediate angiogenesis by transferring genetic material and pro-angiogenic molecules to the damaged areas.^6^ Interestingly, the tissue origin of MSCs has shown to influence the angiogenic potential of the secretome, with different amounts and concentrations of secreted factors in MSCs from different tissue sources. For instance, human adipose tissue derived MSCs (ADSCs) expressed higher levels of insulin-like growth factor 1 (IGF-1), VEGF-D and IL-8 compared with bone marrow derived MSCs (BMSCs), whereas other factor such as VEGF-A, bFGF, SDF-1, and angiogenin were expressed at similar levels.^7^ In the secretome of human Wharton’s jelly derived MSCs (WJSCs) more angiogenesis-related factors were found compared with human BMSCs, inducing superior in vitro microvasculature formation and endothelial cell migration.^8^ Quantitative proteomics revealed an increased angiogenic profile within the secretome of MSC isolated from fetal rather than from adult skin.^9^ Thus, these studies demonstrate that MSCs isolated from different tissue sources show dissimilarities with respect to their transcriptional, proteomic, and functional angiogenic profile. However, so far mainly targeted proteomic approaches have been used with only a limited number of selected angiogenic proteins analyzed. A standardized, systematic comparative analysis of the angiogenic potential of MSC secretomes isolated from different clinically relevant human MSC (hMSC) sources is missing.

The current study aimed to isolate and fully characterize hMSCs secretomes from several donors from human adipose tissue (hADSCs), bone marrow (hBMSCs), and umbilical cord Wharton’s jelly (hWJSCs) in order to systematically compare their angiogenic potential. Importantly, hMSC secretomes were obtained under standardized culture protocols using human platelet lysate (hPL) to provide clinically feasible xenofree culture conditions. A high-resolution, two-dimensional liquid chromatography tandem mass spectrometry (LC-MS/MS) was used to broadly investigate the proteomic profile of hMSC secretomes. Enrichment analyses detected the process of angiogenesis as upregulated.

## Results

### Characterization of hMSCs from different tissue sources

**Fig. 1:**
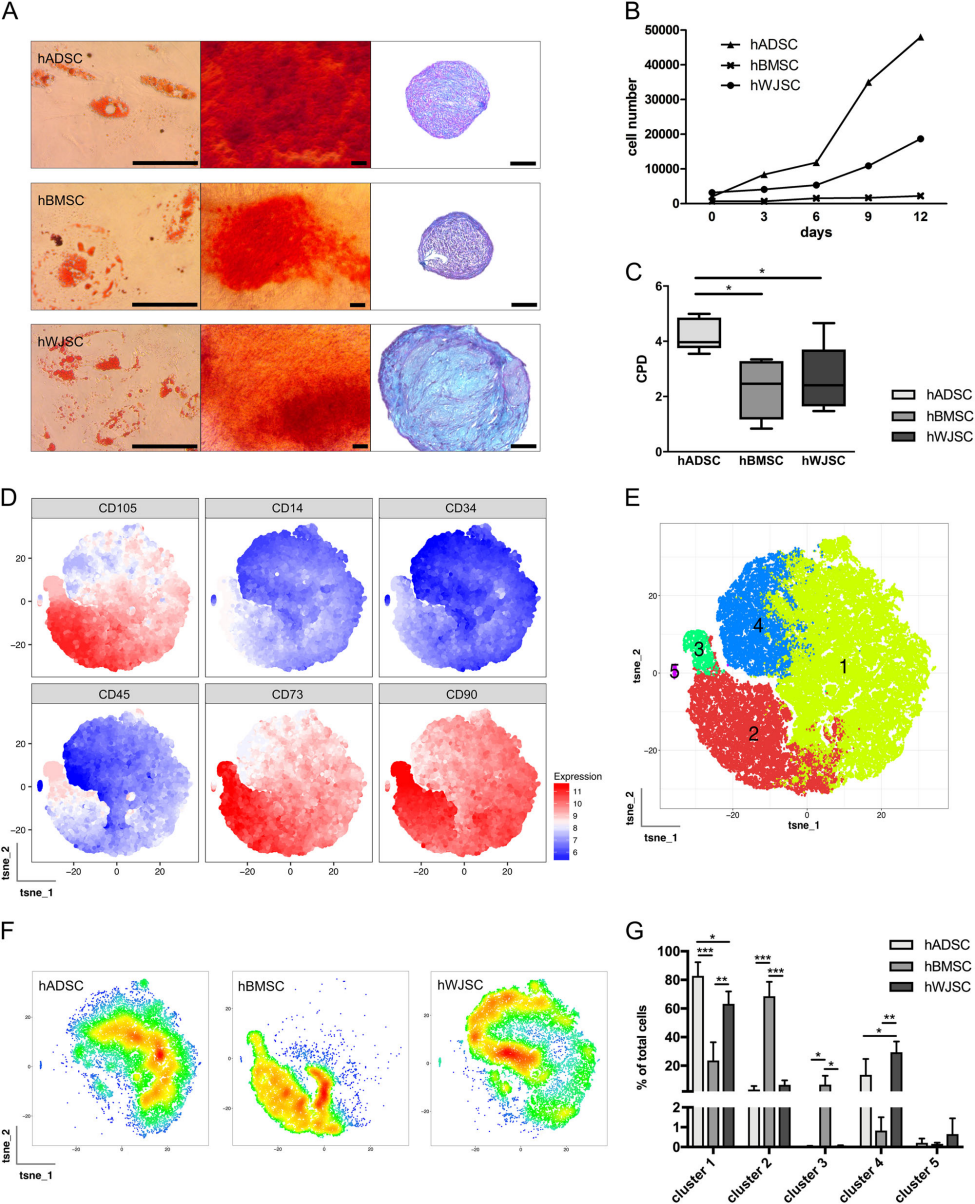
Characterization of hMSC from adipose tissue, bone marrow and umbilical cord Wharton’s jelly. **a** Trilineage differentiation of hMSCs (*n* = 5 per hMSC tissue source) into adipogenic (left; Oil Red O staining), osteogenic (middle; Alzarin Red S) and chondrogenic (right; Alcian Blue PAS) lineages. Scale bar = 100 μm. **b** Proliferation capacities of hMSCs (*n* = 5 per hMSC tissue source) over 12 days, **c** showing the cumulative population doublings of each hMSC source. **d** All hMSCs (*n* = 5 per hMSC tissue source) positively expressed CD73, CD90, CD105, and only a minimal proportion of cells were positive for CD14, CD34 and CD45. Data are visualized by bi-dimensional t-SNE maps generated from multidimensional flow cytometry data. **e** Recorded events were subdivided into 5 clusters by PhenoGraph and FlowSom algorithms according to surface marker expression. The cellular distribution of 5 donors per cell source is **f** visualized with cumulative density plots and **g** quantified accordingly. Bar graphs present mean ± s.d. (*p < 0.05, **p < 0.01, ***p < 0.001; one-way ANOVA and Tukey multiple comparison)

All primary isolated MSC from human adipose tissue (*n* = 5), bone marrow (*n* = 5), and umbilical cord Wharton’s jelly (*n* = 5) from independent donors among the tissue sources were cultured in xenofree proliferation medium based on hPL (for donor characteristics see supplementary Table S1). Multipotent MSC nature of all 15 cell lines was confirmed by trilineage differentiation into adipogenic, osteogenic, and chondrogenic lineages (Fig. 1a; supplementary Fig. S1). No differences were detected in the differentiation potential of hMSC from different tissue sources. hADSCs showed higher proliferation abilities compared with hBMSCs and hWJSCs (Fig. 1b), with significantly higher cumulative population doublings (*p* < 0.05; one-way ANOVA with Tukey multiple correction) (Fig. 1c). Multidimensional data obtained by flow cytometry were processed to generate bidimensional t-SNE maps (Fig. 1d, e; supplementary Fig. S2). The expression profile of each marker within each t-SNE graph is described by cumulative marker expression level plots (Fig. 1d). MSCs from all sources positively expressed CD73, CD90, and CD105, and only a minimal proportion of cells were positive for CD14, CD34, and CD45. The recorded events were subdivided into five sectors by PhenoGraph and FlowSom algorithms (Fig. 1e). Events recorded in cluster 5 are cellular debris. The distribution of the recorded events within the t-SNE maps is visualized by cumulative density plots of each cell source (Fig. 1f). hBMSCs appeared to be significantly more abundant in cluster 2 and 3 and significantly less abundant in cluster 1 and 4, compared with hADSCs and hWJSCs (Fig. 1g). No differences were found for hADSCs and hWJSCs. Cluster 2 and 4 have a higher expression of hMSC markers compared with cluster 1 and 4. These results indicated that the amount of surface proteins was different among the three hMSC sources and that hBMSCs had a more pronounced MSC phenotype compared with hADSCs and hWJSCs.

### hMSC CM harvest and quality assessment

The hMSC CM (*n* = 5 per tissue source) was harvested according to the outlined workflow (supplementary Fig. S3A) at passage 3 to 4. Quality criteria after serum free incubation were defined according to the (I) cellular viability, and the (II) platelet factor 4 (PF-4) concentration in the hMSC CM (supplementary Fig. S3B–D). Furthermore, cellular senescence of hMSCs before serum-free incubation was assessed (supplementary Fig. S4).

**Fig. 2:**
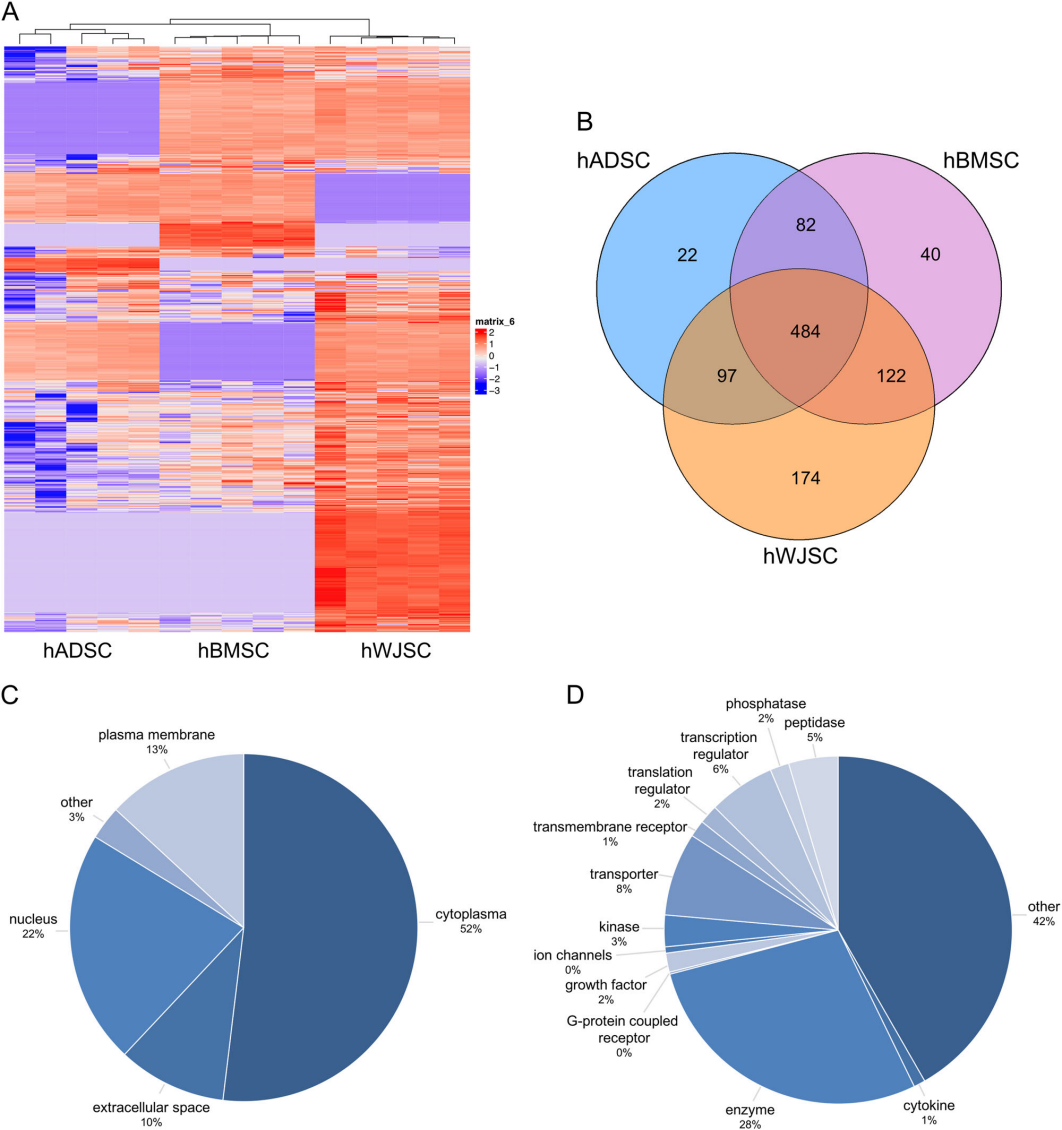
LC/MS-MS analysis of hMSC CM. **a** Heat maps (red = expressed, blue = not expressed) and **b** Venn diagrams represent the total amount of identified proteins in hMSC CM (*n* = 5 per hMSC tissue source), where more exclusive proteins and higher protein intensities were found for hWJSC CM. Identified proteins found in all hMSC CM were characterized on their **d** cellular location and **e** function

Serial dilutions of human platelet lysate allowed the comparison of the initial amount of PF-4 in normal proliferation medium (approximately 1 ug/ml) with the one after the CM harvest procedure (supplementary Fig. S3C). The detection limit of PF-4, consisting of a 1:3200 dilution, was used as a control for LC/MS-MS analysis. No significant differences were detected in cellular viability and PF-4 leftover between the three cell sources by one-way ANOVA (supplementary Fig. S3D). Therefore, the subsequent results obtained with hMSC CM are not biased by differences in serum leftover.

β-galactosidase (β-Gal) activity staining showed a tendency of more β-Gal-positive cells in hADSCs (*p* = 0.09) and hBMSCs (*p* = 0.06) compared with hWJSCs by one-way ANOVA with Tukey multiple comparison, in all cell sources significantly lower to the 4 μM doxorubicin control (supplementary Fig. S4A-B). hADSCs showed enhanced cell cycle activity by significantly upregulated CDKs and corresponding cyclins (see supplementary Fig. S4C for gene panel). This is also supported by significantly higher expression of MYC, E2F1, and TBX3 or lower expression of CDC25C compared to hBMSCs (*p* < 0.01 or *p* < 0.001) and hWJSCs (*p* < 0.01 or *p* < 0.001) (one-way ANOVA with Tukey multiple comparison). Reduced levels of tumor suppressors CDKN2A and CDKN1A demonstrate low activation of effector pathways p16/pRB and p14/p53/p21. Genes associated to DNA damage response and repair, such as ATM, NBN, PCNA, CHEK2, TERF2 were highly upregulated in hADSC compared with hBMSCs (*p* < 0.01 or *p* < 0.001) and hWJSCs (*p* < 0.01 or *p* < 0.001). This means that despite uniform cell culture expansion procedures and consistent cell seeding densities between the three hMSC sources, hADSCs exhibit higher DNA damage response in combination with a stronger proliferative phenotype.

### Mass spectrometry analysis of hMSC CM reveals biological processes related to angiogenesis and endothelial cell function as enriched

**Fig. 3:**
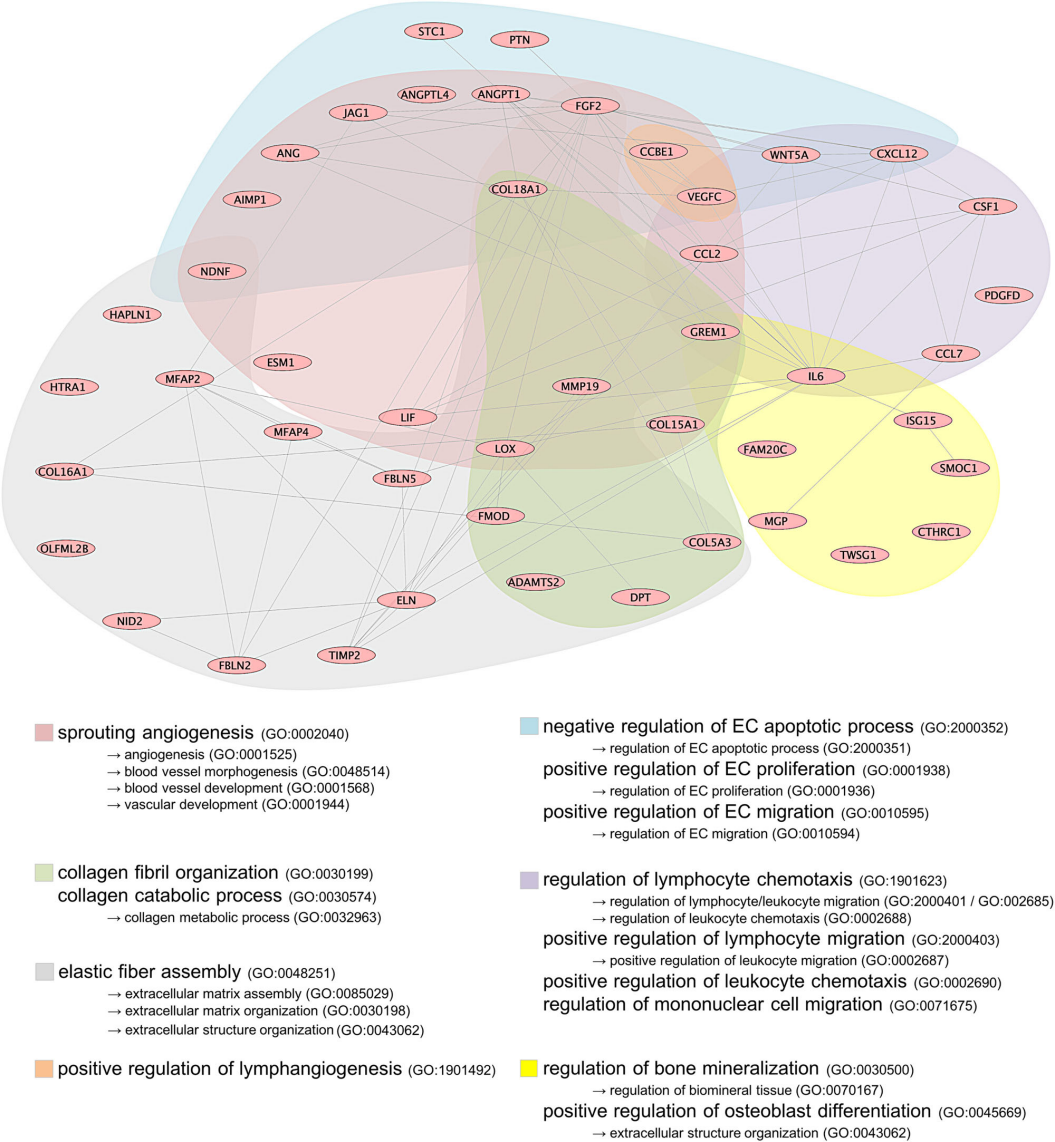
Enriched biological processes in hMSC CM. LC/MS-MS data were analyzed on overrepresented biological processes in the extracellular protein fraction of hMSC CM (*n* = 5 per hMSC tissue source). The most relevant biological processes, their protein-protein interactions and corresponding coloring patterns are displayed

The proteome of hMSC CM was analyzed based on the protein intensities measured by LC/MS-MS (*n* = 5 per tissue source). All proteins detected within the hPL leftover control were excluded from further analysis. A total of 1021 different proteins were identified (List of all proteins: supplement L1). Normalized heat maps revealed a different proteomic profile between the three hMSC CM sources (Fig. 2a). Four hundred and eighty four proteins were common to the three hMSC populations, with 22 proteins being exclusive to hADSC, 40 to hBMSC and 174 to hWJSC (Fig. 2b). Overall higher protein intensities and more exclusive proteins were found in hWJSC. The phylogeny of the heat map confirmed that the CM of hADSC and hBMSC are more related to each other than with hWJSC (Fig. 2a). When looking at the location of proteins, 52% were of cytoplasmic origin, 22% nuclear, 13% from the plasma membrane and 10% from the extracellular space (Fig. 2c); all of them with different functions (Fig. 2d). Processes such as ‘endothelial cell differentiation’, ‘regulation of leukocyte chemotaxis’, ‘extracellular matrix organization’, and ‘angiogenesis’ were shown to be enriched in the overall proteome (Panther overrepresentation list_all: supplement L1), which are particularly of interest for the treatment of ischemic diseases. However, in order to deduce protein functions from gene ontology, additional enrichment analysis of only extracellular proteins was performed (Panther overrepresentation list_extracellular: supplement L1). The most relevant biological processes, their related proteins and interactions are summarized in Fig. 3.

### hWJSC CM retains the highest angiogenic proteome

**Fig. 4:**
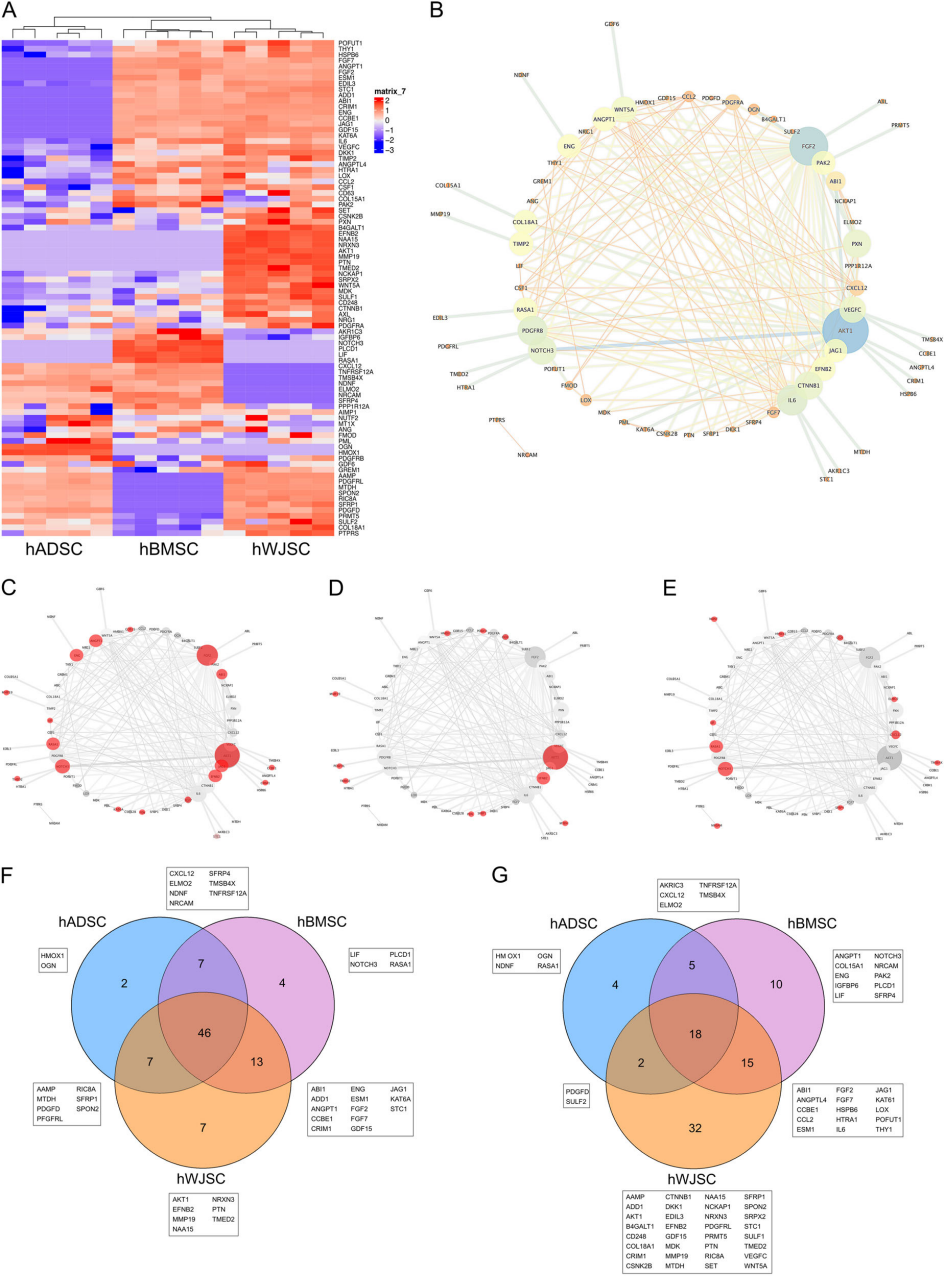
The pro-angiogenic proteome of hMSC CM. **a** Heat map generated based on protein intensities displays hMSC CM proteins involved in angiogenesis (*n* = 5 per hMSC tissue source; red = expressed, blue = not expressed). **b** Interactions of angiogenesis related proteins, where node size/color depends on the amount of protein interactions. Proteins not found in the according CM are displayed in red, demonstrating that central pro-angiogenic proteins are missing in **c** hADSC CM compared to **d** hBMSC CM and **e** hWJSC CM. **f** The protein composition of hMSC CM and their exclusive proteins demonstrate that hBMSC CM and hWJSC CM are closer related compared to hADSC CM; **g** however significantly higher protein intensities by pairwise student t-test were found for hWJSC CM

A particular focus was given to proteins involved in ‘angiogenesis’ and ‘endothelial cell function’, given that ‘sprouting angiogenesis’ (including angiogenesis, blood vessel morphogenesis/development, vascular development) and ‘endothelial cell function’ (including regulation of apoptosis, proliferation and migration) were found to be enriched in the hMSC CM. Eighty six angiogenesis-related proteins were detected in hMSC CM, whereas the phylogeny of the heat maps showed that hBMSC and hWJSC CM are more closely related to each other compared with hADSC CM (Fig. 4a). Forty six angiogenic proteins were common to the 3 hMSC populations, with 2 proteins being exclusive to hADSC, 4 to hBMSC and 7 to hWJSC (Fig. 4f). However, significantly higher protein levels were found in hWJSC CM compared with hBMSC CM and hADSC CM, displayed by Venn diagram (Fig. 4g). Here, the numbers represent the amount of proteins with significantly higher intensities, detected by pairwise student *t*-test between the hMSC groups. All identified proteins were uploaded into String database and evaluated using Cytoscape to define their interactions. Figure 4b displays these protein interactions, where node size/color is based on the ‘BetweennessCentrality’ defined by the combined score of String database. Low values are small sized and orange (color code from low to high: orange–yellow–green–blue). Proteins not being part of any interaction are not further discussed. High ranked proteins are those, which possesses more interactions within the network. AKT1 and FGF2 were highly ranked (blue in Fig. 4b), followed by CTNNB1, IL-6, NOTCH3, PDGFRB, PXN, VEGF-C (green in Fig. 4b) and ANGPT1, COL18A1, EFNB2, ENG, JAG1, PAX2, RASA1, TIMP2, WNT5A (yellow in Fig. 4b). Interestingly, out of this protein selection more crucial angiogenic proteins were missing in the hADSC CM (displayed in red, Fig. 4c), followed by hBMSC CM (Fig. 4d) and hWJSC CM (Fig. 4e). Taken together the LC/MS-MS analysis showed that hWJSC CM retained a more complete pro-angiogenic network with higher concentrations of angiogenesis related proteins.

### hMSC CM induces migration of classical monocytes and enhances macrophage differentiation

**Fig. 5:**
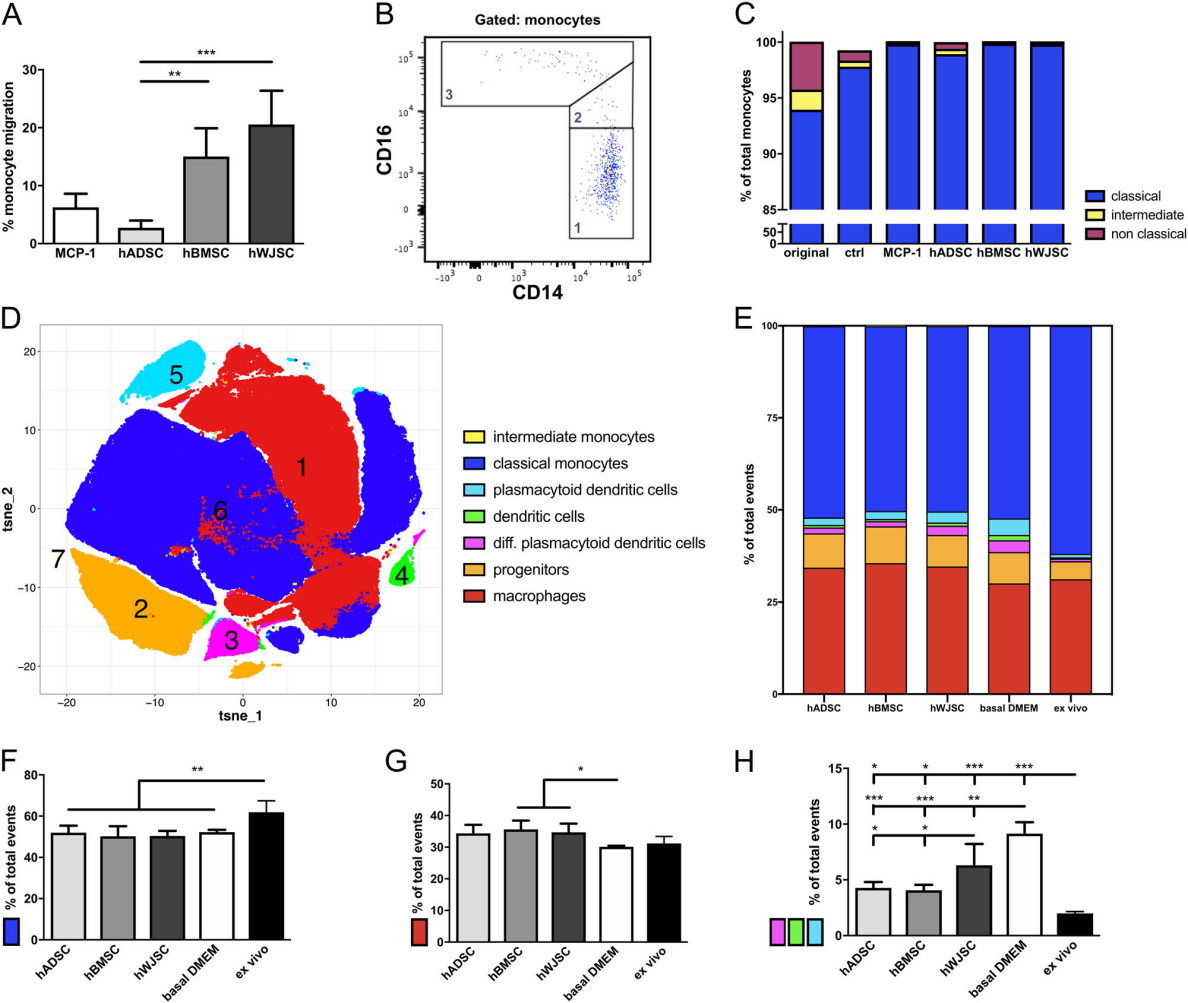
Immune cell response to hMSC CM. **a** hMSC CM (n = 5 per tissue source) revealed enhanced migration of monocytes (*n* = 3 healthy blood donors), with significantly higher migration induced by hBMSC CM and hWJSC CM in comparison to hADSC CM and 5 ng/ml MCP-1 control. **b** Migrated monocytes were characterized by CD14 and CD16 staining, **c** demonstrating that hMSC CM mainly induced migration of classical CD14^++^ CD16^−^ monocytes, but not of intermediate and non-classical monocytes. **d** 7 myeloid cell populations were identified after co-incubation of monocytic Thp-1 cells and hMSC CM (*n* = 5 per tissue source) displayed by a bi-dimensional cumulative vi-SNE map. **e** The percentages of each cell population were monitored, including **f** classical monocytes, **g** macrophages, and **h** all (diff.) (plasmacytoid) dendritic cells. hBMSC CM and hWJSC CM led to a significantly higher monocyte to macrophage differentiation in comparison to hADSC CM and basal DMEM control. Bar graphs present mean ± s.d. (*p < 0.05, **p < 0.01, ***p < 0.001; one-way ANOVA and Tukey multiple comparison)

Immune cells are crucial for the process of angiogenesis, which is also indicated by the analyzed proteomic profile. The immune cell response induced by hMSC CM (*n* = 5 per tissue source) was tested by migration of hPBMCs from three independent healthy donors towards a transwell insert. To assure scalable migration of hPBMCs, the assay was established using different starting amounts of hPBMCs and concentrations of MCP-1 (supplementary Fig. S5A-5B). All results were normalized to the migration induced by basal DMEM medium. hBMSC CM (*p* < 0.01) and hWJSC CM (*p* < 0.001) demonstrated significantly higher monocyte migration when compared with hADSC CM by one-way ANOVA with Tukey multiple comparison, while no differences were detected between the CM of hBMSCs and hWJSC (*p* = 0.07) (Fig. 5a; supplementary Fig. 5C). No significant lymphocyte migration was detected compared with the initial population seeded, supporting the immunomodulatory capacity of hMSC CM (supplementary Fig. S5D). Migrated monocytes were classified using flow cytometry into three subgroups known as classical CD14^++^ CD16^−^ monocytes, intermediate CD14^++^ CD16^+^ monocytes, and non-classical CD14^dim^ CD16^+^ monocytes (Fig. 5b). The original seeded hPBMC population consisted of 93.50 ± 2.94% classical, 2.04 ± 0.62% intermediate, and 4.51 ± 2.56% non-classical monocytes of total monocytes analyzed (Fig. 5c). After migration mainly classical CD14^++^ CD16^+^ monocytes were detected.

Next, monocytic Thp-1 cells were analyzed after co-incubation with either hMSC CM (*n* = 5 per tissue source), basal DMEM medium (negative control), or original ex vivo Thp-1 medium (positive control). The results obtained by multicolor flow cytometry were computed using dimensionality reduction and clustering algorithms. Seven cell populations were identified in cumulative vi-SNE maps based on the expression levels of key surface markers (Fig. 5d; supplementary Fig. S6; supplementary Fig. S7).^10^ Each of the myeloid population was monitored after co-incubation and the percentages of events per population (Fig. 5e) and density distributions analyzed (supplementary Fig. S8). We observed a significant decrease in the classical monocyte population when the cells were co-incubated with hMSC CM (*p* < 0.01) or basal DMEM only (*p* < 0.01) by one-way ANOVA with Tukey multiple comparison (Fig. 5f). However, only co-incubation with hBMSC CM (*p* < 0.05) and hWJSC CM (*p* < 0.05) induced a significant increase of macrophages in the overall population compared with basal DMEM (Fig. 5g), emphasizing that only these two CM led to a significantly higher monocyte to macrophage differentiation. All hMSC secretomes significantly inhibited dendritic cell maturation compared with basal DMEM (*p* < 0.01 or *p* < 0.001) (Fig. 5h). Based on these functional results the proteins detected by LC/MS-MS related to phagocyte, lymphocyte, and granulocyte regulation, activation and chemotaxis were summarized in supplementary Fig. S9. Significantly higher inflammation-related protein levels were found in hWJSC CM compared with hBMSC and hADSC CM.

### hMSC CM shows angiogenic potential in vitro and in vivo

**Fig. 6:**
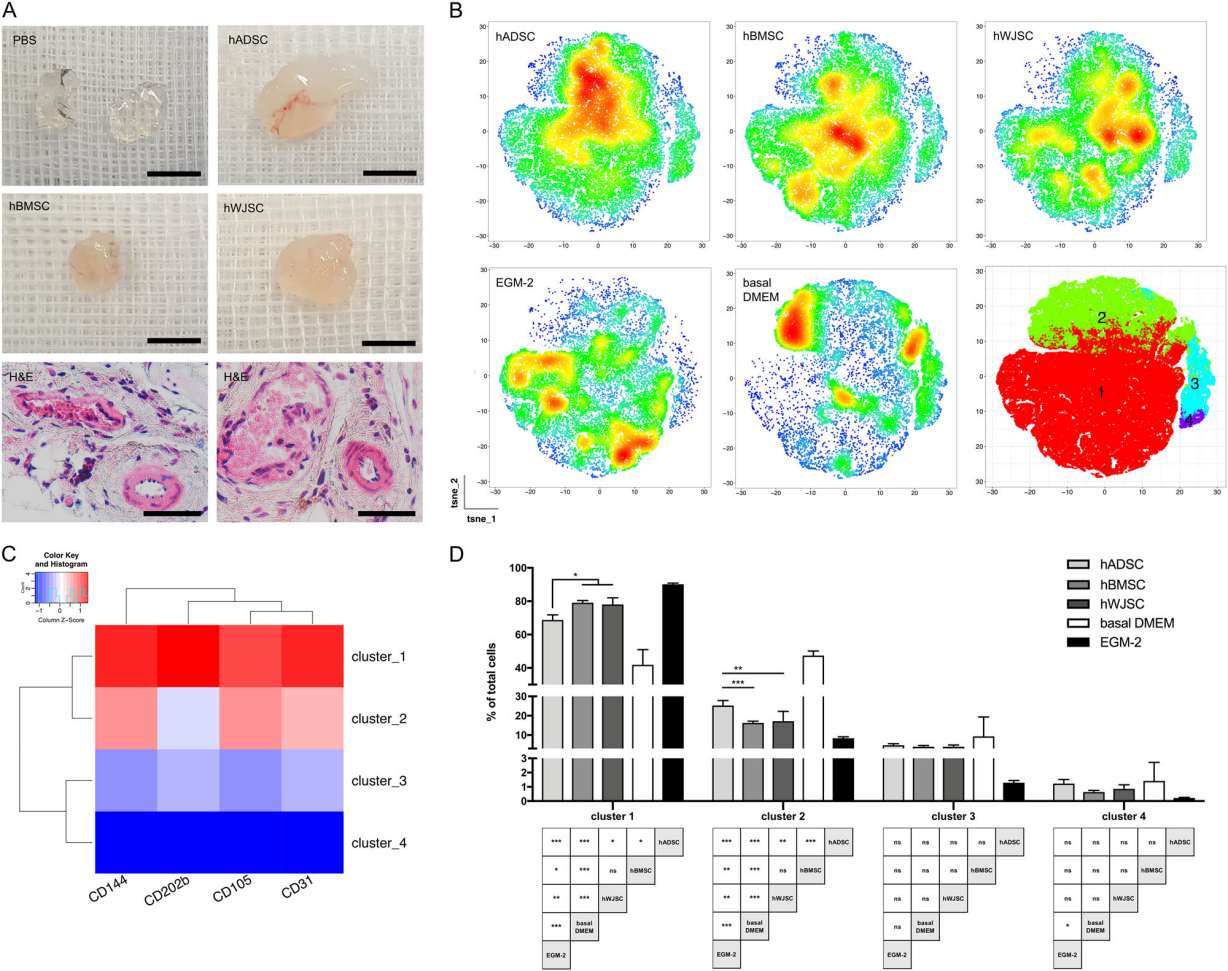
hMSC secretome is pro-angiogenic in vitro and in vivo. **a** In vivo Matrigel plug assay demonstrated significantly increased vascularization when using hMSC CM compared to basal medium only. Scale bar = 0.5 cm / H&E = 100 μm. **b**–**d** Stimulation of HUVEC with hMSC CM (*n* = 5 per tissue source) induces expression of angiogenesis related markers, including CD31, CD105, CD202b, and CD144. **b** The density distribution of cells was dependent on the co-incubation medium. Clustering flow cytometry data defined 4 different clusters, **c** each with a distinct expression profile. **d** A significantly higher shift towards the pro-angiogenic cluster 1 was found when HUVEC were co-incubated with hBMSC CM and hWJSC CM. Bar graphs present mean ± s.d. (*p < 0.05, **p < 0.01, ***p < 0.001; one-way ANOVA and Tukey multiple comparison)

As a proof of concept, the pro-angiogenic potential of hMSC CM detected by LC/MS-MS was evaluated in vivo by performing a subcutaneous Matrigel plug assay in mice (Fig. 6a). Compared with controls, all hMSC secretomes enhanced vascularization of Matrigel plugs. This was confirmed macroscopically and histologically by H&E staining (Fig. 6a).

To analyze the pro-angiogenic potential of hMSC CM in vitro, surface marker expression of CD202b, CD105, CD144, and CD31 were analyzed in HUVECs after co-incubation with either hMSC CM (*n* = 5 per tissue source), basal DMEM medium (negative control), or original EGM-2 medium (positive control). Clustering multidimensional flow cytometry data using FlowSOM defined 4 different cell populations according to the expression levels of angiogenesis related surface markers (Fig. 6b, c; supplementary Fig. S10). From the surface antigen expression levels these populations represent different stages of endothelial cells with a different angiogenic-related phenotype. Cluster 1 represents a pro-angiogenic HUVEC phenotype with high expression of all analyzed surface antigens, followed by cluster 2 with an intermediate phenotype and clusters 3 and 4 with a low phenotype. The density distribution of the cells was dependent on the medium HUVECs were co-incubated with (Fig. 6b). Vi-SNE maps from each biological sample used to generate cumulative vi-SNE maps are displayed in supplementary Figs. S11-S12. A significant shift towards cluster 1 was found when the cells were co-incubated with hMSC CM compared with basal DMEM only. A significantly higher shift towards the pro-angiogenic cluster 1 was found when HUVEC were co-incubated with hBMSC (*p* < 0.05) and hWJSC CM (*p* < 0.05) compared with hADSC CM by one-way ANOVA with Tukey multiple comparison (Fig. 6d). In cluster 2 a higher percentage of total cells were found for hADSC CM compared with hBMSC CM (*p* < 0.001) and hWJSC CM (*p* < 0.01) (Fig. 6d). These results demonstrate that hBMSC CM and hWJSC CM induce a more angiogenic HUVEC phenotype in vitro compared with the pro-angiogenic induction triggered by hADSC CM. The cellular phenotype of HUVEC after co-incubation with hBMSC CM and hWJSC CM closer resembled the phenotype obtained by treatment with original EGM-2 medium.

## Discussion

The current study demonstrates how the tissue origin influences the proteomic profile of the hMSC secretomes. We observed that biological processes related to the regulation of leukocyte chemotaxis, endothelial cell function, and angiogenesis are overrepresented in the proteomic profile of hMSC CM. However, our results indicated that the composition and concentration of angiogenic proteins vary between different hMSC CM sources and ultimately influence the provoked functional responses.

hBMSC CM and hWJSC CM retained a higher angiogenic profile when compared with the hADSC CM. hADSC CM showed a lower amount of angiogenesis related proteins and expressed most of them to a significantly lower level. Interestingly, hADSC CM lacked central angiogenic proteins, such as AKT1 and FGF2, compared with hBMSC CM and hWJSC CM. AKT1 has shown to promote angiogenesis-related functions of BMSCs in vivo^11^ and its overexpression in MSCs from amniotic fluid^12^ and bone marrow^13,14^ supported the repair of ischemic cardiac tissue in vivo. Also, FGF2 is known as a potent inducer of angiogenesis.^15^ FGF2 addition has shown to enhance the long-term angiogenic efficacy of transplanted hADSCs into ischemic tissue^16^ and induce the production of MSC-derived extracellular vesicles for vessel stabilization.^17^ Interestingly, differences in the angiogenic proteome between hBMSC CM and hWJSC CM were not primarily based on protein composition, but rather on the amount of the detected proteins itself. Thirty-seven percent of all angiogenic proteins were measured in significantly higher amounts within the hWJSC CM compared with hADSC CM and hBMSC CM. Previous comparative studies have also shown that hWJSC CM possesses more secreted factors involved in angiogenesis compared with hBMSC CM.^8^ Ribeiro et al. proposed that hBMSC CM might be most potent to reduce oxidative stress, while hWJSC CM and hADSC CM seem more beneficial in the protection against excitotoxicity.^18^ It was also suggested that hWJSCs express more stemness and growth-related genes, whereas hBMSCs express more genes involved in skeletal development.^19^

In accordance with the proteomic profile, weaker responses towards hADSC CM were functionally confirmed in vitro. hBMSC CM and hWJSC CM significantly enhanced the migration of classical monocytes and led to significantly higher monocyte to macrophage differentiation. The recruitment of circulating monocytes to post-capillary venules has shown to be essential for microvascular growth.^20^ Monocyte-derived macrophages regulate several steps in the process of angiogenesis, including the secretion of angiogenesis-promoting paracrine factors, the remodeling of the extracellular matrix for capillary sprouting and the interaction with other cell types such as endothelial cells, pericytes, and vascular smooth muscle cells.^20^ It has been suggested that the monocyte/macrophage infiltration and angiogenesis are spatially correlated^20^ and that infiltrating cells regulate the patterning of the local ECM degradation, consequently influencing the distribution of neovessel formation and their branch points.^21^ hBMSC CM and hWJSC CM also induced a significantly stronger angiogenic phenotype of co-cultured endothelial cell. Angiogenesis initiation by hMSC CM is of particular interest for the treatment of diseases related to ischemia or insufficient/abnormal vessel growth. Here, we showed as a proof of concept that application of hMSC CM can promote vascularization of in vivo implanted Matrigel plugs in mice. However, further studies are necessary to directly compare the angiogenesis potential of the three hMSC CM in vivo, including the amount of newly formed sprouts, as well as their maturation state.

Minimal criteria for characterizing multipotent MSCs defined in 2006 by the International Society for Cellular Therapy (ISCT)^22^ do not consider potential proteomic and functional differences between hMSC isolated from different tissue sources. Trilineage differentiation and MSC surface markers failed to sufficiently phenotype the cellular composition and biological function of hMSCs in accordance with previous studies.^23^ These results also highlight the ongoing discussion about the need for optimized MSC characterization parameters, with the ultimate goal to enable comparison across multiple research fields and clinical studies involving hMSCs.^23^ In fact, currently used ISCT criteria produce heterogeneous, non-clonal cultures of stromal cells containing multipotent stem cells, committed progenitors, and differentiated cells.^24^ We are therefore still far from defining a molecular signature for hMSCs from different tissue sources, also considering different percentages of stem cells among donors within the heterogenous hMSC population. Importantly, patient variability due to general characteristics (i.e., age, gender etc.) or underlying health status (i.e., comorbidities, nonsteroidal anti-inflammatory drugs etc.) cannot be excluded in the presented study despite consistent isolation and characterization procedures. MSCs have been isolated from independent donors among the different tissue sources. Although a direct comparison of MSCs derived from all three tissue sources of the same donor would have been desirable, this was impossible due to ethical and logistical reasons.

A major challenge of in vitro secretome studies lies in discriminating proteins that are truly MSC-secreted from those that are artifacts derived from cell culture media components (such as serum proteins) or derived from non-physiological stresses affecting the starving cells. In this regard, hMSC CM harvest procedures allow for the attachment of serum proteins that can later be released into the hMSC CM.^25^ This contamination with serum proteins might have been neglected in many previous hMSC secretomes-based studies, which may have led to misleading scientific conclusions. Therefore, the presented study for the first time defined remaining serum proteins by the detection of PF-4 and used a serial dilution of it as a control for the LC/MS-MS analysis. However, proteins identified in any of the PF-4 controls were not included into the analysis. Therefore, some proteins might have been missed by this procedure as they can be derived from both, hMSCs and hPL. In particular low concentration proteins released from hMSCs will overlap with proteins in the hPL control. Further, intracellular proteins can be detected in the hMSC CM due to cellular death or leakage. There is now evidence that some of these proteins can also be released via nonclassical pathways as vesicles and exosomes and exert extracellular functions that seem to be different from their intracellular role.^25^ Considering non-physiological stresses during secretomes harvest, it has been shown that factors released from apoptotic MSCs can contribute to the regeneration of ischemic tissues,^26,27^ but that senescence also induces changes in the composition of the secretome and therefore might influence MSC physiology.^28^ Sun et al. highlighted the beneficial immunomodulatory roles of apoptotic multipotent stem cells by demonstrating that combined therapy with apoptotic and healthy ADSCs is superior to either alone.^27^ However, on the other side replicate senescence in later passage hMSCs has been associated with reduced clinical outcome in patients with graft-versus-host disease.^29^ We showed that despite consistent cell culture procedures, hWJSC exhibit lower senescence-associated β-Gal activity and hADSCs exhibit higher DNA damage response in combination with a proliferative phenotype. This hADSCs phenotype is supported by the fact that overexpression of oncogenes MYC and E2F1 are known to initiate a first transient hyperproliferation phase with a subsequent DNA damage response finally resulting in cellular senescence.^30,31^ We believe that the activation of DNA damage response has been initiated in hADSC, but that the threshold for reaching cellular senescence and cell cycle arrest has not been reached so far. These differences might have influenced the angiogenic potential of hADSCs, however its causative role cannot be defined with the current data set.

Overall the presented study describes the cellular origin and function of all detected proteins and did not only focus on the extracellular subset. We believe that the complete secretome needs to be described in order to understand protein interactions and in vivo functionality. Unlike pharmaceutical treatments that deliver a single agent, the hMSC secretome provides several stimulatory and inhibitory bioactive factors at variable concentrations that might sustain physiological kinetics in the local microenvironment. In ischemic heart diseases, single cytokine therapy trials have not met expectations suggesting that processes such as angiogenesis may need simultaneous orchestration of multiple factors at different concentrations to act synergistically.^32^ High concentrations of single factors can even lead to the formation of aberrant and leaky vessels, hypotension and tumor angiogenesis.^33^ Furthermore, the establishment of clinical treatments based on hMSC secretome has clear advantages for clinical translation and applicability compared with currently used autologous or allogenic cell therapies. In particular when manufacturing costs and logistics, handling, safety and regulatory aspects of hMSC secretome are considered. The unlimited supply of the hMSC secretome off-the-shelf without the need for harvesting autologous patient tissue would represents a major advantage. So far, there is only a limited number of first in men studies and clinical trials initiated using MSC secretomes in the field of wound healing,^34^ graft-versus-host disease,^35^ hair loss,^36,37^ multiple sclerosis,^38^ and alveolar bone regeneration.^39^ However, all applications have shown safety without any severe adverse reactions reported and point towards a potential clinical use in the future.

In conclusion, we have systematically compared hMSC from adipose tissue, bone marrow, and umbilical cord Wharton’s jelly in order to define which cell source possesses the highest potential for angiogenesis. A xenofree approach towards generating hMSC CM and enabling safe clinical translation has been established. hMSC CM from different tissue origins showed significant functional and proteomic differences with regards to angiogenesis. Proteomic results indicate that hWJSC CM might represent the most potent hMSC source for therapeutic angiogenesis induction as hWJSC CM showed higher concentrations of angiogenesis related proteins. Even if the functional responses between hBMSC CM and hWJSC CM were comparable, the amount of hBMSC obtained from invasive bone marrow puncture is limited and susceptible to aging factors, involving the accumulation of cellular damage, cell death, and senescence. hWJSCs can be obtained from healthy, young tissue with minimal ethical concerns. Therefore, the noninvasive harvest, its fetal origin and unlimited supply as leftover material, supports the highest therapeutic value of hWJSC secretomes for angiogenesis induction. In adult patients, our results indicate that hBMSC CM has a higher potential for angiogenesis when compared with the same amount of hADSC CM. These differences may thus have implications on the selection of hMSCs in future clinical studies.

## Methods

### hMSC isolation from different human tissues

hMSC from human adipose tissue (*n* = 5), bone marrow aspirate (*n* = 5), and umbilical cord Wharton’s Jelly (*n* = 5) were isolated from independent donors among the tissue sources (supplementary Table 1 for hMSC donor characteristics) and cultured in a humidified incubator at 5% CO_2_ at 37 °C using standard proliferation medium consisting of high-glucose DMEM (Sigma, Switzerland) supplemented with 10% human platelet lysate (hPL), 1% Glutamax (Gibco, USA), and an antibiotic-antimycotic solution (Sigma, Switzerland). hPL was obtained from platelet apheresis collection of donors compliant with local blood donation regulations (Center for Blood Donation Zurich, Switzerland). Medium changes were done every third day and passaging was performed using Accustase (Innovative Cell Technologies Inc., USA).

Adipose tissue (*n* = 5) obtained from surgically performed liposuction procedure after written informed consent from the donors was used for the isolation of hADSCs approved by the cantonal ethics committee Zurich, Switzerland (KEK-ZH-2010-0476). Adipose tissue was digested using collagenase type A (Roche Diagnostics GmbH, Switzerland) at 37 °C for 1 h under constant gentle motion. The sample was filtered using a nylon cell strainer with 70 μm mesh size (BD Bioscience, USA) and separated by histopaque-1077 density gradient centrifugation (Sigma, Switzerland) for 30 min at 600 × *g*. The interphase was plated in standard proliferation medium consisting of high-glucose DMEM (Sigma, Switzerland) supplemented with 10% human platelet lysate (hPL), 1% Glutamax (Gibco, USA), and an antibiotic-antimycotic solution (Sigma, Switzerland) for cellular attachment and growth.

Human bone marrow aspirate (*n* = 5) was collected in sterile heparinized tubes (BD Bioscience, USA) after written informed consent from the patients approved by the cantonal ethics committee Bern, Switzerland. For isolation of hBMSCs the aspirate was separated by histopaque-1077 density gradient centrifugation for 25 min at 800 × *g*. Subsequently, the mononuclear cell layer was collected, washed, and plated in standard proliferation medium.

Human umbilical cords (*n* = 5) were obtained after full-term births with written informed consent of the donors approved by the cantonal ethics committee Zurich, Switzerland (KEK-ZH-2009-0095), and processed for isolation of hWJSC. Wharton’s jelly (perivascular and intermediate Wharton’s jelly region) was dissected from the umbilical cord and cut into small pieces (1 mm^2^). After letting the tissues adhere to the culture plate, standard proliferation medium was gently added. Tissue pieces were removed after a first cellular outgrowth could be detected and cells were harvested for subsequent passaging.

### hMSC characterization

Multilineage differentiation was assessed by inducing differentiation to osteocytes, adipocytes, and chondrocytes as previously described.^40^ After 3 weeks, cells were fixed in 4% paraformaldehyde/PBS and stained with (I) 2% Alzarin Red S (Sigma, Switzerland), (II) 3 mg/ml Oil Red O (Sigma, Switzerland) or III) Alcian Blue PAS.

Proliferation was assessed at day 3, 6, 9, and 12. Cells were fixed with methanol for 10 min (Sigma, Switzerland) and stained with 0.1% crystal violet (Artechemis, Switzerland) for 5 min. After washing and air-drying, absorbed crystal violet was solubilized with Na-deoxycholate (Sigma, Switzerland) while being heated at 60 °C for 10 min. The absorbance was recorded at 550 nm using a standard ELISA reader Synergy HT (Bio TEK, USA). Quantitative data was obtained using a standard curve with serial dilutions. Generation times (GT) were calculated as previously described.^41^

hMSCs were characterize on commonly used surface markers CD14, CD34, CD45, CD73, CD90, and CD105 by flow cytometry. 2.5 × 10^5^ hMSCs were stained for 20 min at 4 °C according to standard procedures with the antibodies listed in supplementary Table S2. Fifty thousand events were acquired using a LSR Fortessa (BD Bioscience, USA) and the data sets were analyzed by FlowJo software (Tree Star, Inc., USA) gating viable and single cells (for gating strategy see supplementary Fig. S13). Bidimensional t-SNE maps were generated from multidimensional flow cytometry data sets of viable and single cells using the open source R platform and the Cytokit package (http://bioconductor.org). Five thousand events were randomly selected and clustered using PhenoGraph algorithm (*k* = 40). PhenoGraph arranges all events in the multidimensional space according to similarities in surface marker expression levels and allowed to plot the data in two dimensions using bi-dimensional visual stochastic neighbor embedding (vi-SNE) graphs. We used the clustering by FlowSOM and generated t-SNE graphs to analyze and detect subset differences in MSC marker expression profile.^42^ Each recorded cell is positioned according to surface marker similarities in separate regions.

### hMSC conditioned media harvest and quality assessment

For hMSC secretomes harvest, defined as conditioned media (CM), all hMSC lines (passage 3 to 4) were cultured until 80% confluence in standard proliferation medium, washed three times with PBS and starved in serum-free conditions (basal medium only) for 16 h. Collected CM was filtered using a 0.2 μm filter, concentrated using centrifugal filter units with a 3-kDA cutoff at 4 °C (Amicon Ultra Centrifugal filters, Millipore, USA) and stored at −80 °C for further analysis. Aliquots of stored hMSC CM were derived from 1 × 10^6^ hMSCs in 0.2 ml. Exact number and viability of starved cells was obtained using a NucleoCounter NC-200 (CHemoMetec, Denmark). Histochemical staining of β-galactosidase (β-Gal) activity (Sigma, Switzerland) was used to quantify the percentage of senescent cells by the number of blue, β-Gal-positive cells after incubation under standard culture conditions and 4 μM doxorubicin (Sigma, Switzerland). In addition, a qRT-PCR of senescence associated genes was performed using standard conditions on a QuantStudio 7 flex real-time PCR system (Quiagen, Switzerland and Applied Biosystems, Thermofisher, USA). GAPDH served as housekeeping gene to quantify relative stemness expression using the ΔCT threshold cycle method and calculated with 2^−ΔCT^. Serum leftover of the primarily added hPL was assessed by quantitative evaluation of platelet factor 4 (PF-4), a cytokine uniquely released from activated platelets, using a multiplex particle-based flow cytometric cytokine assay.^43^ PF-4 served as a surrogate marker for the remaining hPL concentration in order to exclude biases due to differences in the post-starvation hPL content between different MSC sources.

### Mass spectrometry analysis

Proteins of hMSC CM derived from 1 × 10^6^ cells (not concentrated) were retrieved using 5 μl Strata Clean resin beads per ml of CM (Agilent Technologies, USA) and further processed for in-gel digestion as described in Gazdhar et al.^44^ An aliquot of 5 μl peptide extract was analyzed by LC-MS/MS on an EASY-nLC1000 chromatograph connected to a QExactive HF mass spectrometer (ThermoFisher Scientific) using a Pepmap100 Trap C18 300 mm × 5 mm (Thermo Fisher Scientific) and a C18 separation column (3 μm, 100 Å, 75 μm × 15 cm, Nikkyo Technos, Tokyo, Japan) by applying a 40 min gradient as previously described.^45^ LC-MS/MS data was processed with MaxQuant (version 1.5.4.1) using default settings for peak detection, strict trypsin cleavage rule allowing for upto three missed cleavages, variable oxidation on methionine, deamidation of asparagine and glutamine, and acetylation of protein N-termini with strict carbamidomethylation of cysteines. Match between runs was used within each sample group with a retention time window of 1 min. The fragment spectra were interpreted with the SwissProt Homo sapiens database (version 2016_04) accepting only protein identifications with at least two razor peptides at a 1% false discovery rate (FDR) cutoff.

For MS data analysis, peptide feature intensities were group-wise median normalized and the intensities of the three most intense peptides summed to the protein intensity. When a group had at least one positive identification, missing values were imputed from the low end of the LOG2 transformed intensity distribution from each individual sample using Perseus (version 1.5.5.3) as suggested by Lazar et al.^46^ Proteins identified in any of the three platelet controls were not included for pairwise Students *T*-tests and a multi sample ANOVA tests, applying a permutation-based FDR of 1% as an acceptation criteria for differentially expressed proteins and to correct for multiple testing. Cellular function and location was defined using the Ingenuity Pathway Analysis (Qiagen). Evaluation of overrepresented GO biological processes (complete) of proteins was performed using Panther (www.pantherdb.org) and a Fisher’s Exact with FDR multiple test correction. To display protein interactions selected proteins were uploaded into String database (www.string-db.og) and evaluated using Cytoscape (www.cytoscape.org).

### hPBMC migration towards hMSC CM

Heparinized human peripheral blood was obtained from healthy donors (*n* = 3) after informed written consent approved by the cantonal ethics committee Zurich, Switzerland (KEK-ZH-2014-0430). Human peripheral blood mononuclear cells (hPBMCs) were isolated according to standard protocols by ficoll density gradient centrifugation (Histopaque-1077; Sigma, Switzerland). Migration of 3 × 10^6^ hPBMCs in 300 μl basal medium supplemented with 0.5% fetal calf serum (FCS) (Sigma, Switzerland) towards the lower compartment containing either (I) 0.1 ml hMSC CM + 0.5% FCS (*n* = 5 per tissue source), (II) basal medium + 0.5% FCS or (III) 5 ng/ml monocyte chemoattractant protein 1 (MCP-1) + 0.5% FCS, was evaluated through a PET membrane with 3 μm pores (Corning, USA) after 3 h. The number of migrated cells was defined by counting cells over 1 min on a FACSCanto II (BD Bioscience, USA). Monocyte subpopulations were differentiated by staining with CD14, CD16, and propidium iodide (PI) (supplementary Table S2; supplementary Fig. S13).

### Thp-1/hMSC CM co-incubation

The monocyte phenotype after co-incubation with hMSC CM was analyzed using the human monocytic leukemia cell line (Thp-1; Sigma, Switzerland). Thp-1 cells were cultured in suspension using xVivo15 medium (Lonza, Switzerland) and the medium was changed every 2–3 days. Thp-1 cells were co-incubated for 16 h with either (I) hMSC CM (*n* = 5 per hMSC group), (II) xVivo15 (n = 5) or (III) basal DMEM (*n* = 5). 0.2 ml hMSC CM diluted 1:3 in basal DMEM was used per 100’000 Thp-1. After co-incubation, floating and attached Thp-1 were harvested using Accutase and stained 20 min at 4 °C with CD11b, CD14, CD16, CD36, and SRA-I (supplementary Table S2). All events were acquired using a LSR Fortessa and the data sets were analyzed by FlowJo software gating viable and single cells (for gating strategy see supplementary Fig. S13). Bidimensional t-SNE maps were generated from multidimensional flow cytometry data (all events included) and PhenoGraph algorithm employed.

### In vivo matrigel plug assay

The in vivo pro-angiogenic potential of hMSC CM was assessed performing the Matrigel plug assay in C57BL/l mice (female, 7-week-old) divided into four groups: control (*n* = 5; basal medium), hADSC CM (*n* = 5), hBMSC CM (*n* = 5) and hWJSC CM (*n* = 5). For each condition 0.2 ml of medium was mixed with 0.3 ml phenol red-free, growth factor reduced basement membrane matrix (Corning, USA). Media were injected subcutaneously into the left and right dorsal flank of mice (*n* = 10 per group). After 14 days, mice were sacrificed and the matrigel plugs were harvested. Matrigels were analyzed macroscopically and histologically. All animal experiments were approved by the Cantonal Ethics Committee (State Veterinary Office of the Canton of Zurich, Switzerland; No. 105/2016).

### HUVEC/hMSC co-incubation

Endothelial cells were harvested from human umbilical cords following written informed consent of the patients approved by the cantonal ethics committee Zurich, Switzerland (KEK-ZH-2009-0095). Briefly, HUVEC were dissociated from the venous lumen by incubating a collagenase solution (2 mg/ml collagenase type A, Roche Diagnostics GmBH, Switzerland) for 30 min in a humidified incubator at 37 °C and obtained in endothelial growth medium (EGM-2; Lonza, Switzerland).

After several passages, HUVEC were starved in endothelial basal medium (EBM-2; Lonza, Switzerland) with 1% FCS for 4 h and co-incubated for 16 h with either (I) hMSC CM + 0.5% FCS (*n* = 5 per hMSC group), (II) EGM-2 (*n* = 5) or (III) basal DMEM + 0.5% FCS (*n* = 5). 0.2 ml hMSC CM diluted 1:3 in basal DMEM was used per 100,000 HUVEC. Harvested HUVEC were stained 20 min at 4 °C with the following surface antibodies CD31, CD105, CD144, and CD202b (supplementary Table S2). 50,000 events acquired on a LSR Fortessa were analyzed by FlowJo software gating viable and single cells (for gating strategy see supplementary Fig. S13). Bidimensional t-SNE maps were generated (5000 events) and PhenoGraph algorithm applied.

### Statistical analysis

Quantitative data are presented as mean ± standard deviation (GraphPad Prism; Graph Pad Software, Inc.). For statistical comparison One-Way ANOVA with Tukey multiple comparison test was used. Saphiro-Wilk normality test proved that the datasets were normally distributed. Bar graphs present Mean ± Standard Deviation (s.d.). *P*-values < 0.05 were considered statistically significant (*p < 0.05, **p < 0.01, ***p < 0.001).

### Reporting Summary

Further information on experimental design is available in the Nature Research Reporting Summary linked to this article.

## Supplementary information

Supplementary Material

Reporting Summary

Supplementary Protein List L1

## Data Availability

The authors declare that all data supporting the findings of this study are available within the article and its supplementary material files, or from the corresponding author upon reasonable request.

## Supplementary information

**Supplementary information** accompanies the paper on the *npj Regenerative Medicine* website (10.1038/s41536-019-0070-y).
