# Supplementary material for: Proteomic analysis of human mesenchymal stromal cell secretomes: a systematic comparison of the angiogenic potential
Source: NPJ Regen Med. 2019 Apr 16;4:8. doi: 10.1038/s41536-019-0070-y (PMC6467904; doi:10.1038/s41536-019-0070-y)
Supplement: Supplementary file 1 — Supplementary Material [file 41536_2019_70_MOESM1_ESM.pdf]

Supplementary Material

Proteomic analysis of human mesenchymal stromal cell secretomes: a systematic comparison of the angiogenic potential

Debora Kehl[1], Melanie Generali[1], Anna Mallone[1], Manfred Heller[2,3], Anne-Christine Uldry[2,3], Phil Cheng[4], Benjamin Gantenbein[3,5,6,7], Simon P. Hoerstrup[1,5,8,9*], Benedikt Weber[1,5,8,10*]

* These senior authors contributed equally to this manuscript

[1] Institute for Regenerative Medicine (IREM), University of Zurich, Zurich, Switzerland
[2] Mass Spectrometry and Proteomics Core Facility, University of Bern, Bern, Switzerland
[3] Department for BioMedical Research (DBMR), University of Bern, Bern, Switzerland
[4] Department of Dermatology, University Hospital Zurich, Zurich, Switzerland
[5] Center for Applied Biotechnology and Molecular Medicine (CABMM), University of Zurich, Zurich, Switzerland
[6] Tissue and Organ Mechanobiology, Institute for Surgical Technology and Biomechanics, University of Bern, Switzerland
[7] Department for Orthopaedics & Traumatology, Insel University Hospital, University of Bern, Bern, Switzerland
[8] Zurich Center for Integrative Human Physiology (ZHIP), University of Zurich, Zurich, Switzerland
[9] Wyss Zurich, University of Zurich and ETH Zurich, Zurich, Switzerland
[10] Skin and Endothelium Research Division (SERD), Department of Dermatology, Medical University of Vienna, Austria

Content Synopsis:

Supplementary Figure S1-S13

Supplementary Table S1-S2

source	*sample*	*sex*	*year of birth*
adipose tissue	hADSC (A2)	female	1970
adipose tissue	hADSC (A5)	female	1982
adipose tissue	hADSC (A6)	female	1971
adipose tissue	hADSC (A7)	female	1942
adipose tissue	hADSC (A8)	female	1932
bone marrow	hBMSC (B1)	male	1942
bone marrow	hBMSC (B2)	female	1936
bone marrow	hBMSC (B5)	female	1950
bone marrow	hBMSC (B7)	female	1941
bone marrow	hBMSC (B8)	female	1956
Wharton's jelly	hWJSC (W1)	female (child)	1980 (mother)
Wharton's jelly	hWJSC (W2)	male (child)	1980 (mother)
Wharton's jelly	hWJSC (W4)	male (child)	1971 (mother)
Wharton's jelly	hWJSC (W5)	female (child)	1972 (mother)
Wharton's jelly	hWJSC (W6)	male (child)	1985 (mother)

Supplementary table S1: hMSC donor characteristics

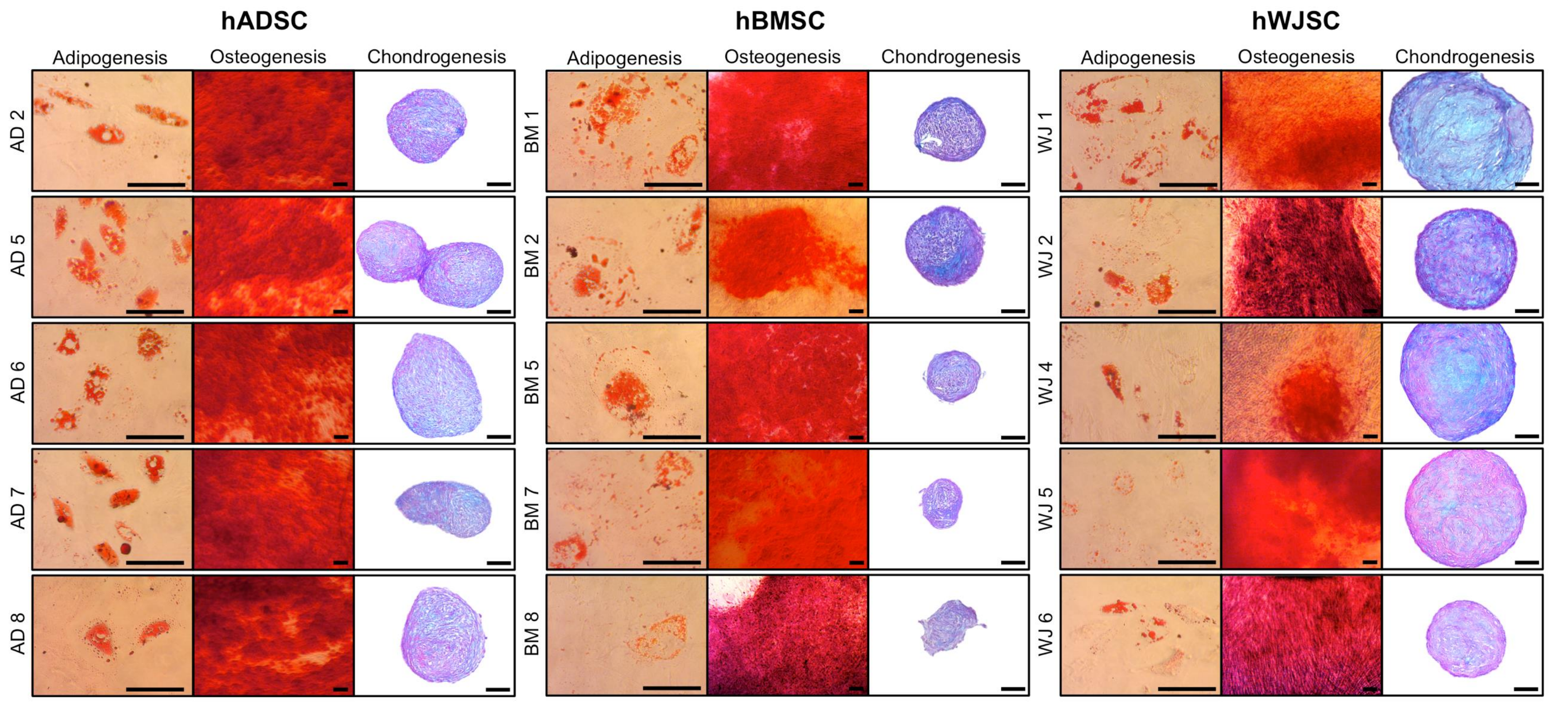

Supplementary figure S1: Multilineage differentiation potential of hMSCs from adipose tissue, bone marrow and umbilical cord Wharton's jelly. Differentiation of hADSC, hBMSC and hWJSC (n=5 per tissue source) into adipogenic, osteogenic and chondrogenic lineages is visualized with Oil Red O, Alzarin Red S and Alcian Blue PAS staining. Scale bar = 100um.

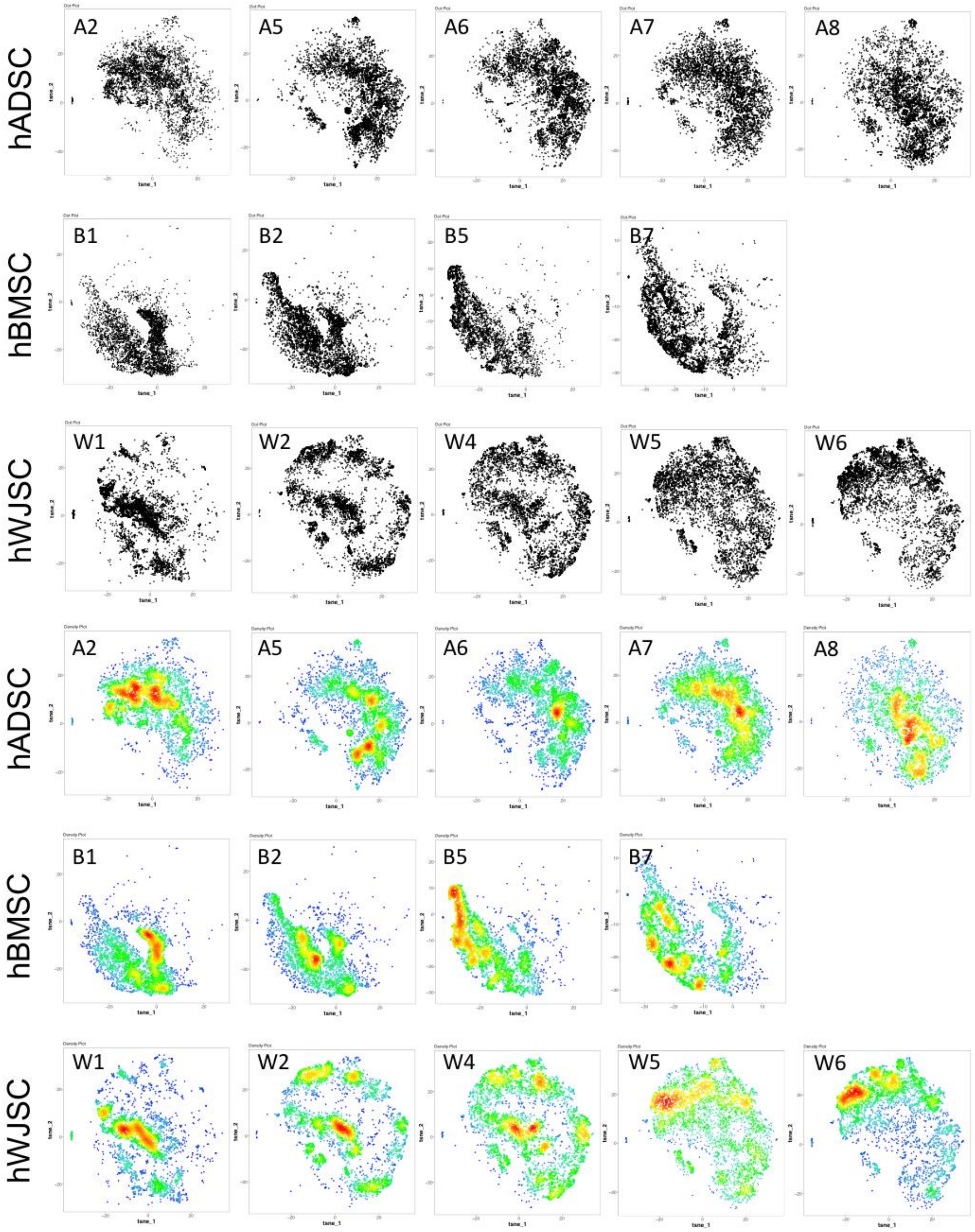

Supplementary figure S2: vi-SNE maps from hMSC from adipose tissue, bone marrow and umbilical cord Wharton's jelly. Vi-SNE and density maps from each particular hMSC donor used to assemble cumulative vi-SNE and density maps (see figure 1D-G).

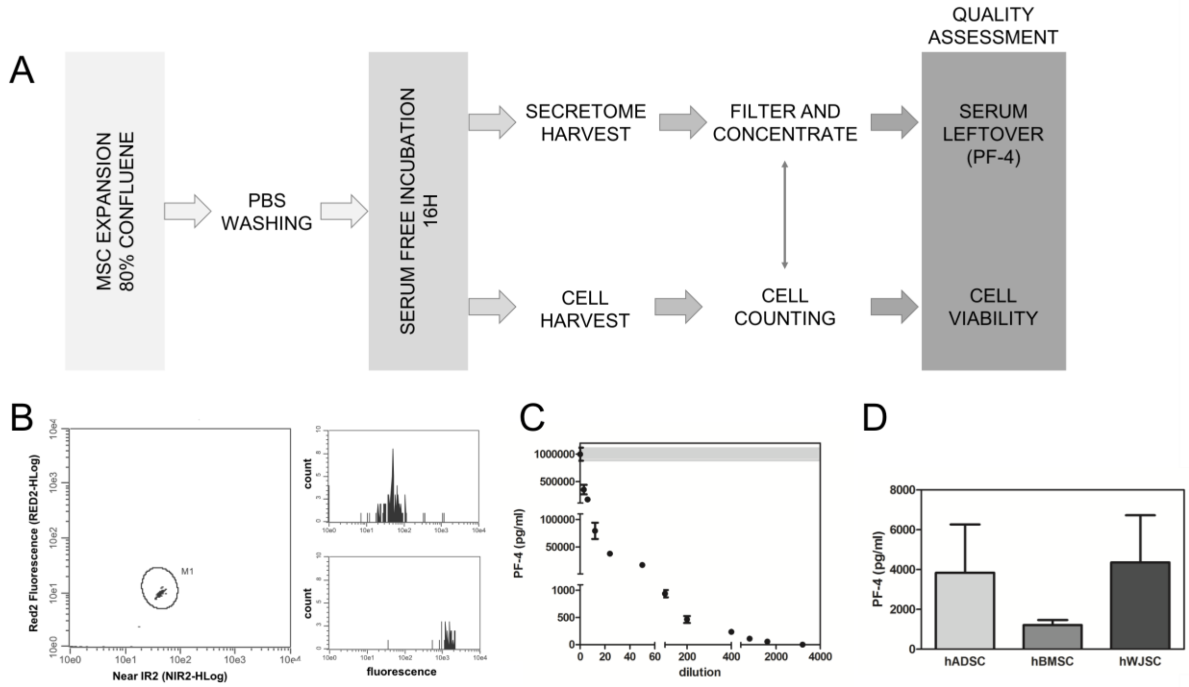

Supplementary figure S3: hMSC CM harvest. (A) Workflow for hMSC CM harvesting. Quality criteria were defined to analyze possible human platelet lysate (hPL) leftover after serum free incubation. (B-C) For the detection of serum contaminations, a serial dilution of 10% hPL in basal DMEM was performed using multiplexed particle-based flow cytometric cytokine assay. (B) The left panel displays the gating of platelet factor-4 (PF-4) and its quantification was done according to the right low and high standards. (C) At a dilution of 3200 no PF-4 was detected, which demonstrates the baseline for LC/MS-MS analysis and subsequent functional assays. (D) Concentrated hMSC CM (n=5 per hMSC tissue source) revealed no significant differences in PF-4 leftover between the three hMSC tissue sources. Bar graphs present mean ± s.d. (*p<0.05, **p<0.01, ***p<0.001; one-way ANOVA and Tukey multiple comparison)

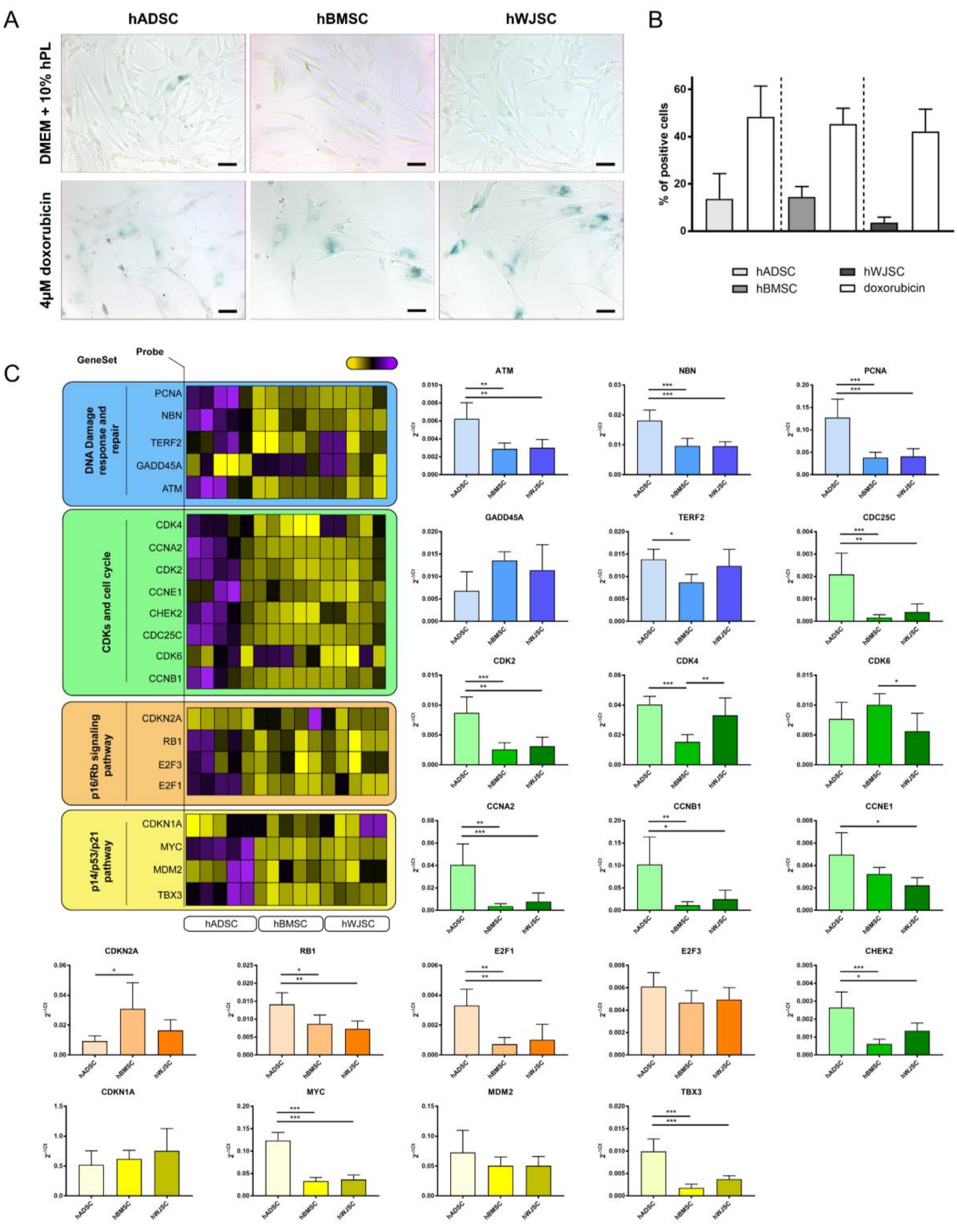

Supplementary figure S4: The senescence-associated phenotype of hMSCs. (A) β-galactosidase (β-Gal) activity staining of hMSCs (n=5 per hMSC tissue source; scale bar = 200µm) at passage 3 to 4 in standard culture medium and after 4µM doxorubicin (B) demonstrate a higher percentage of blue β-Gal-positive cells in hADSCs and hBMSC compared to hWJSCs. (C) Gene expression data of gene sets involved in DNA damage response and repair (blue), CDKs and cell cycle (green), p16/Rb signaling pathway (orange) and p14/p53/p21 pathway (yellow). Quantitative PCR data are displayed as $2^{-\Delta CT}$. hADSCs exhibit higher DNA damage response in combination with a stronger proliferative phenotype. Bar graphs present mean ± s.d. (*p<0.05, **p<0.01, ***p<0.001; one-way ANOVA and Tukey multiple comparison)

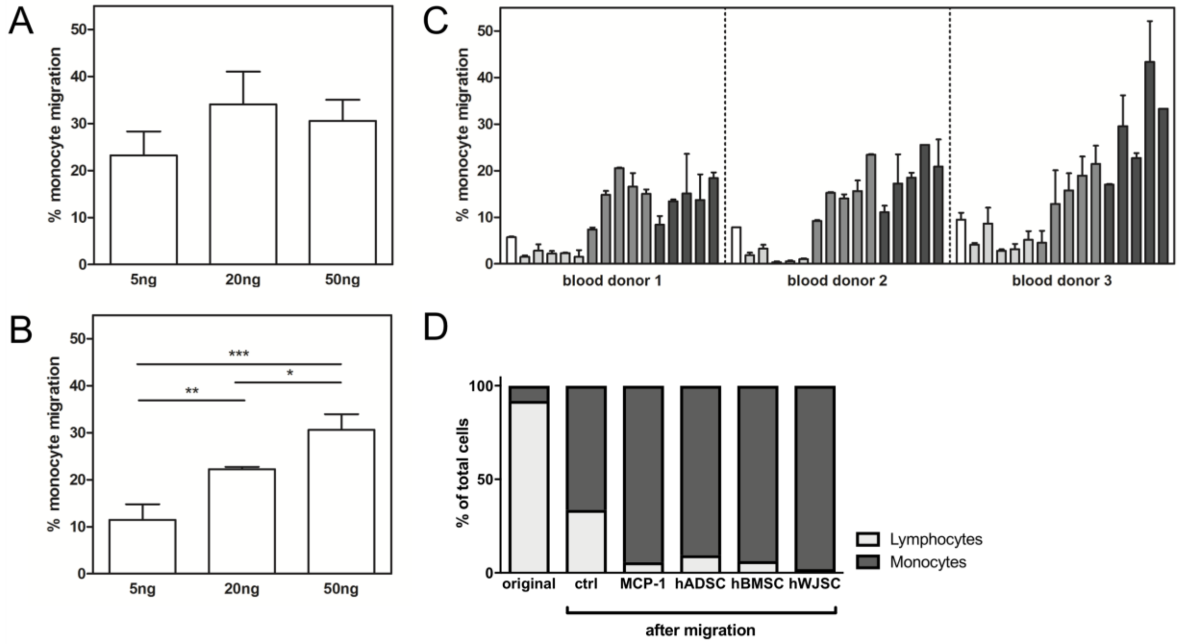

Supplementary figure S5: *In vitro* transmigration of hPBMCs to hMSC secretome. Concentration dependent migration of hPBMCs (n=3 healthy blood donors) through 3µm pores of a transwell insert was evaluated after 3h co-incubation with 5ng/ml, 20ng/ml and 50ng/ml MCP-1. (A) At a starting amount of $1x10^6$ hPBMC no concentration dependent monocyte migration was found, (B) when compared to a starting amount of $3x10^6$ hPBMCs. (C) Results of the three individual blood donors used to evaluate monocyte migration towards hMSC CM (n=5 per hMSC tissue source). (D) After migration towards hMSC CM no significant lymphocyte migration was detected. Bar graphs present mean ± s.d. (*p<0.05, **p<0.01, ***p<0.001; one-way ANOVA and Tukey multiple comparison)

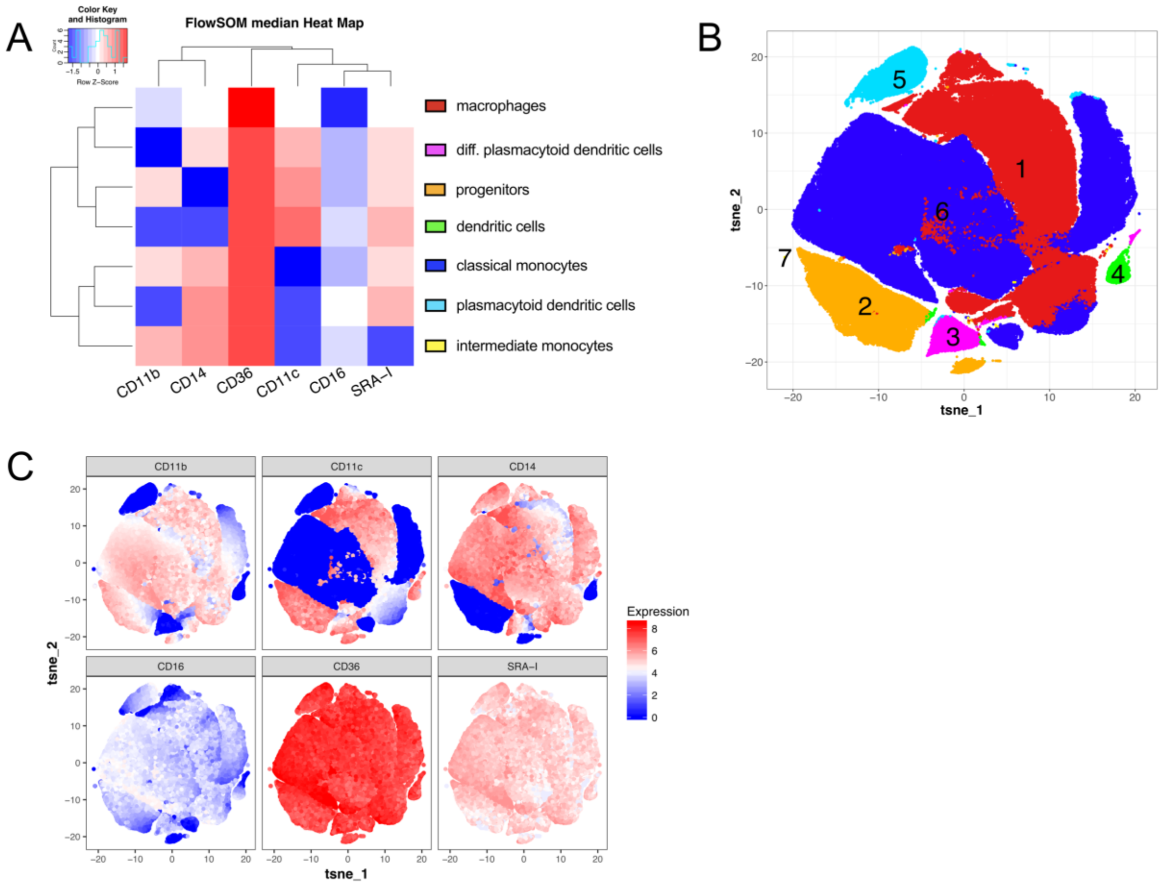

Supplementary figure S6: Expression of key surface markers to differentiate myeloid subsets. 7 cell populations were identified using vi-SNE according to the expression levels of key surface markers after hMSC CM co-incubation (n=5 per hMSC tissue source). (A) The heat map shows the median fluorescence intensity of each of the 6 analyzed surface markers. (B) Based on the expression pattern indicated by the heat map each cluster could be allocated to one specific myeloid subset consisting of 1) macrophages, 2) progenitors, 3) differentiated plasmacytoid dendritic cells, 4) dendritic cells, 5) plasmacytoid dendritic cells, 6) classical monocytes, and 7) intermediate monocytes (see figure 4D-E). (C) The expression profile of the subset can also be visualized by cumulative marker level expression plots.

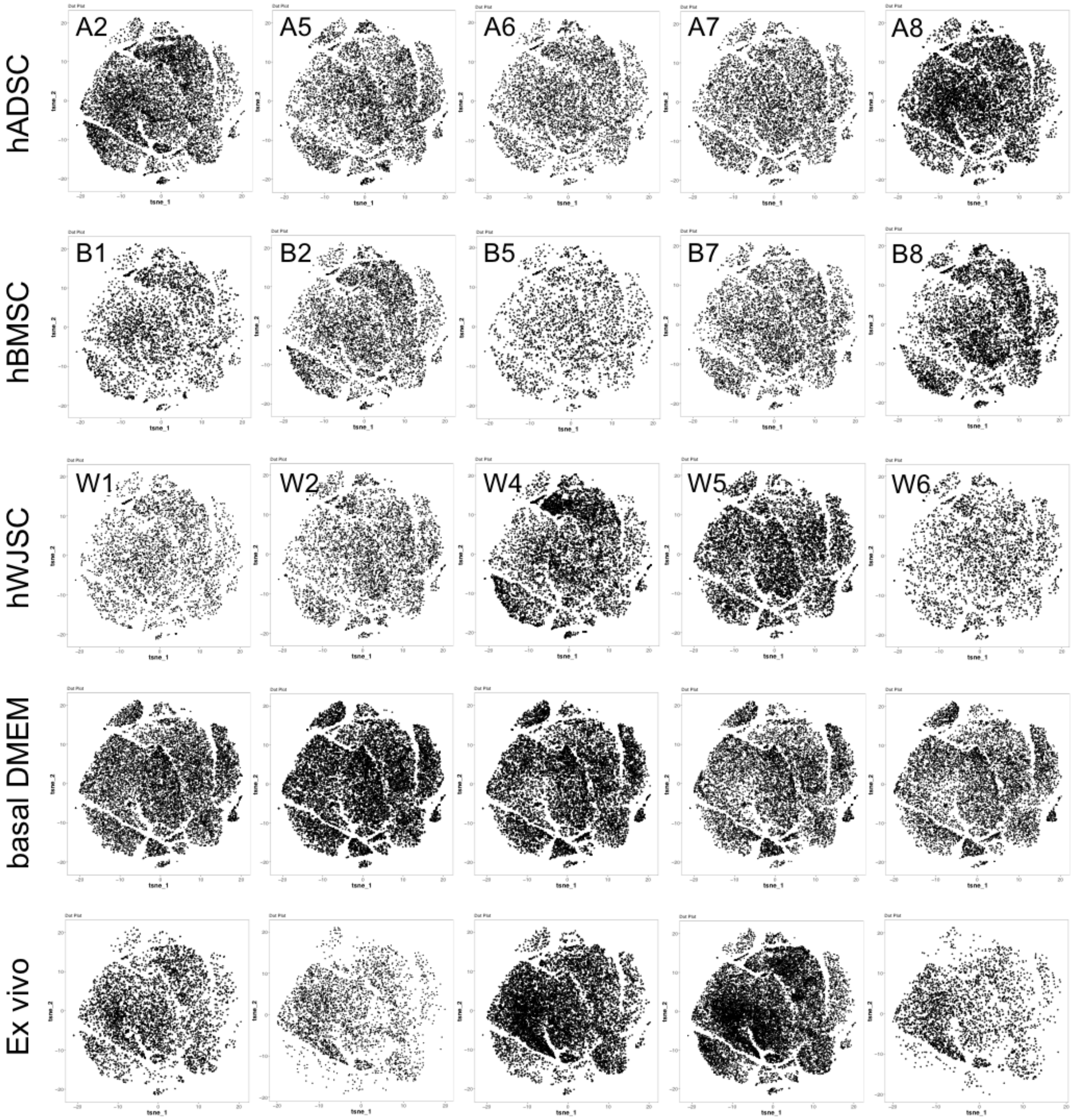

Supplementary figure S7: vi-SNE maps from thp-1 cells after hMSC CM co-incubation. Vi-SNE maps from each biological replicate (n=5 hMSC tissue source) used to assemble cumulative vi-SNE maps (see figure 4D).

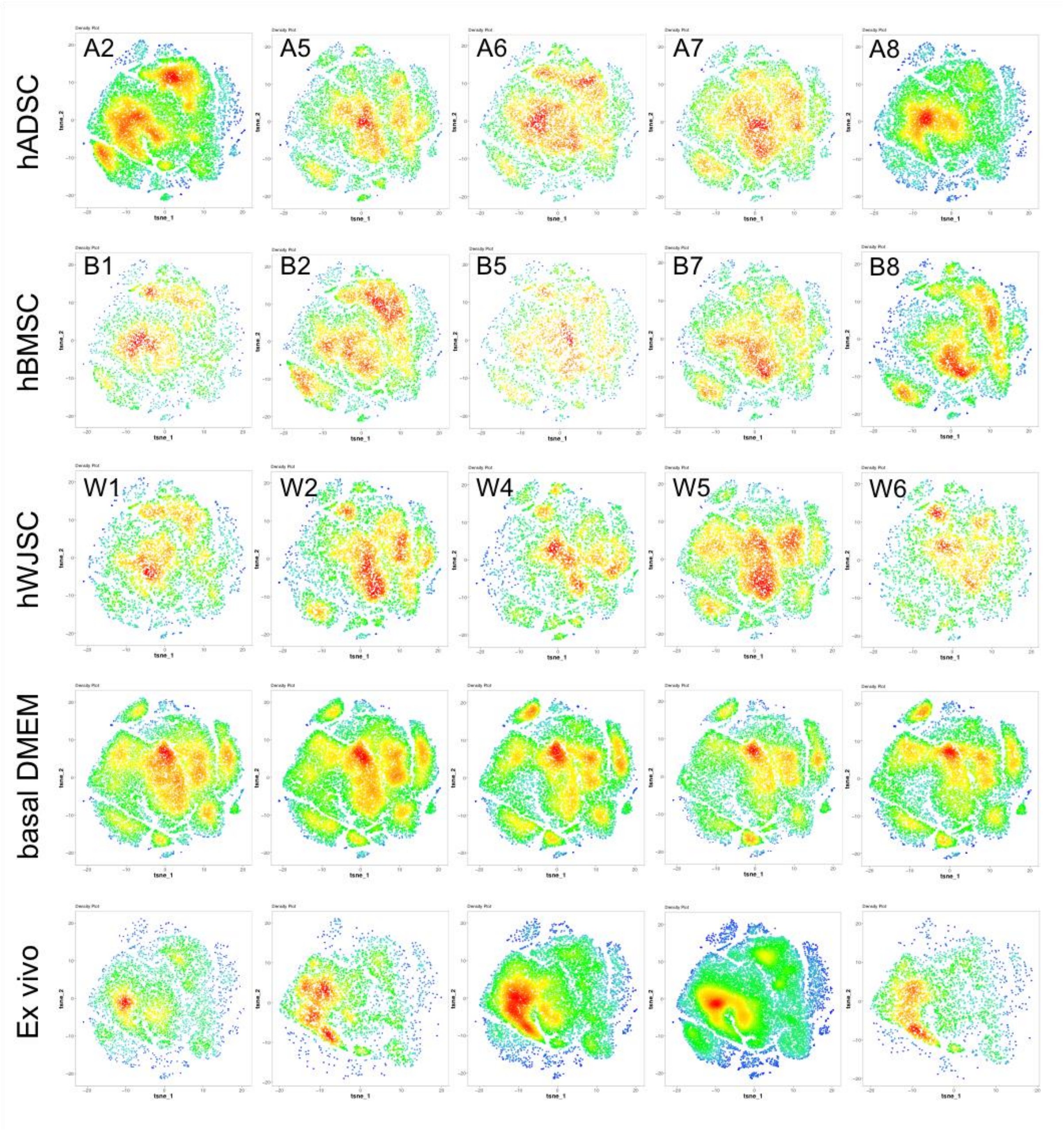

Supplementary figure S8: Density maps from thp-1 cells after hMSC CM co-incubation. Density vi-SNE maps from each biological replicate (n=5 hMSC tissue source) used to assemble cumulative vi-SNE maps (see figure 4D).

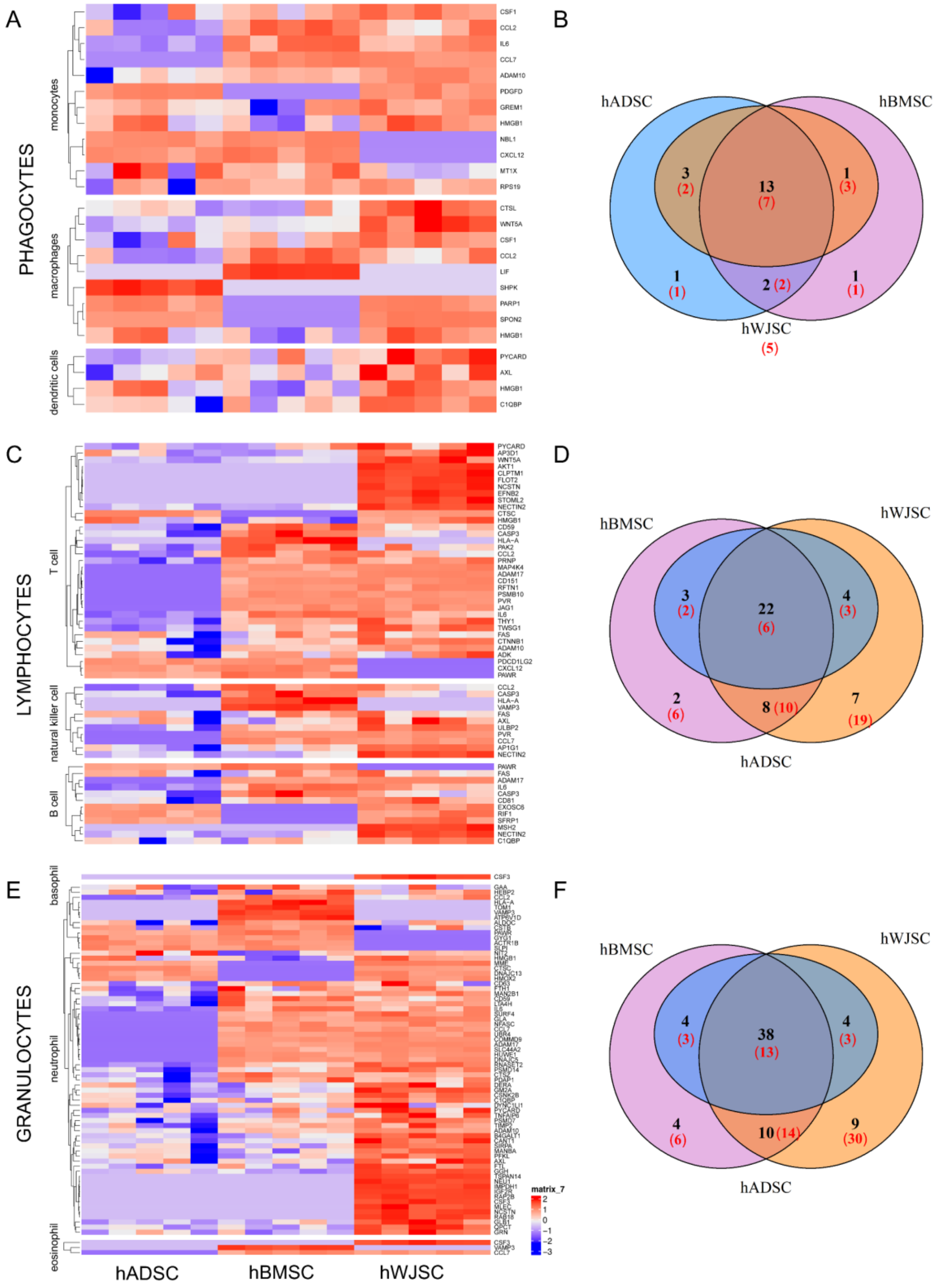

Supplementary figure S9: The inflammatory proteome of hMSC CM. Heat maps generated based on protein intensities measured by LC/MS-MS display proteins involved in (A) phagocyte, (C) lymphocyte and (E) granulocyte regulation, activation and chemotaxis (n=5 per hMSC tissue source, red = expressed, blue = not expressed). Venn diagrams display proteins of each of the three hMSC cell sources involved in (B) phagocyte, (D) lymphocyte and (F) granulocyte function. Significantly higher protein levels by pairwise student t-test are displayed by the corresponding red number.

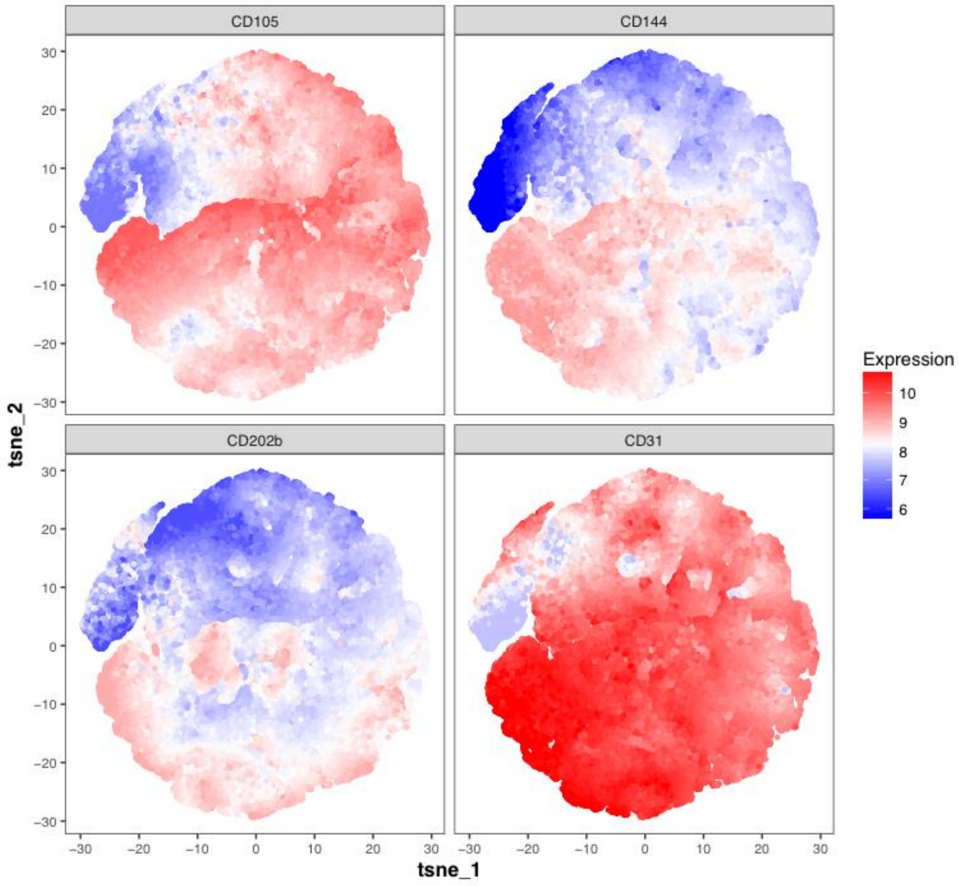

Supplementary figure S10: Cumulative marker level expression plots from HUVEC after hMSC CM co-incubation. Overview of the expression levels of CD105, CD144, CD202b and CD31 in generated bi-dimensional tsne maps (n=5 per hMSC tissue source). Each recorded cell by flow cytometry is positioned according to surface marker similarities in separate regions. These data were used to define clusters (see figure 5C).

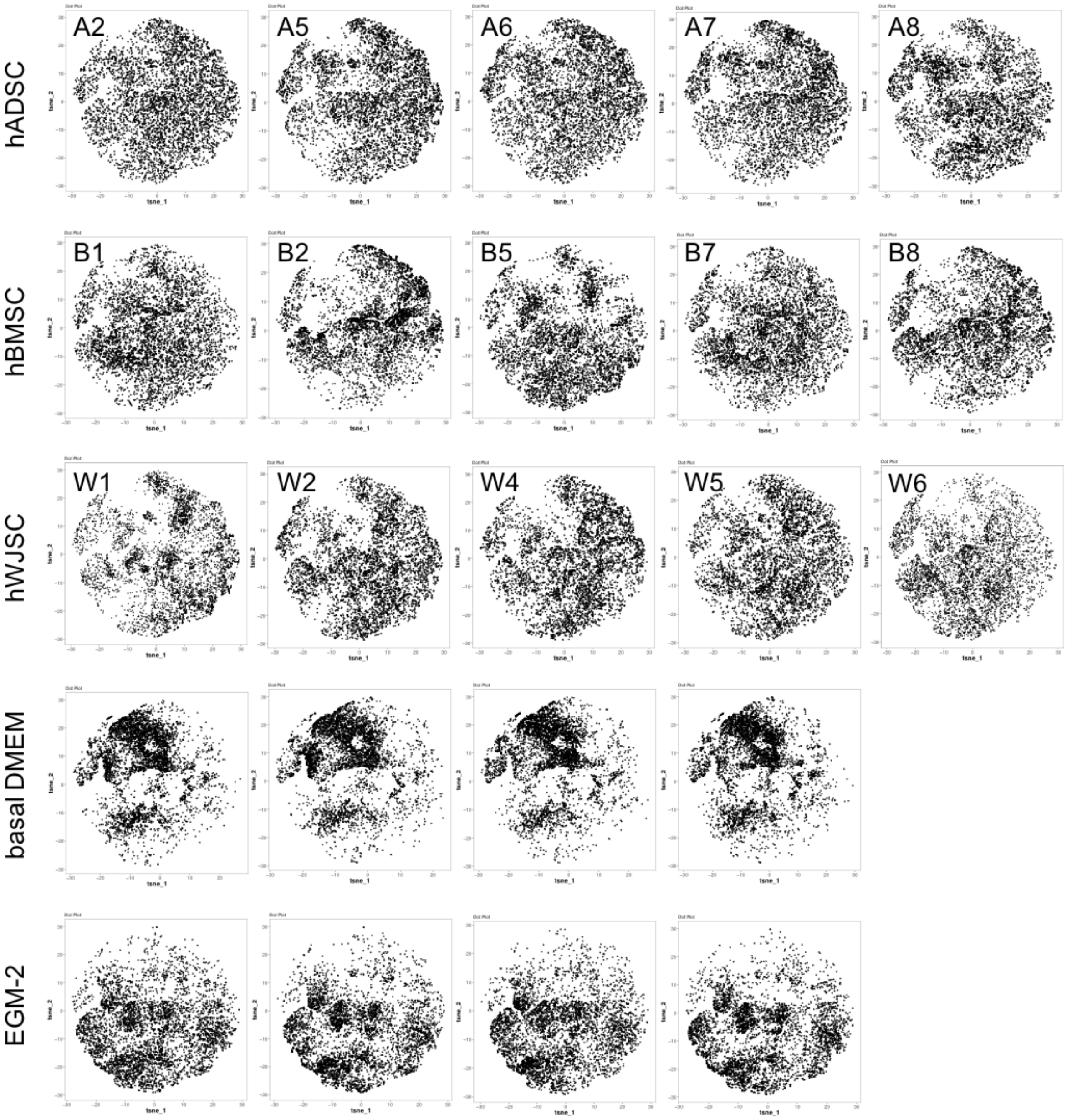

Supplementary figure S11: vi-SNE maps from HUVEC after hMSC CM co-incubation. Vi-SNE maps from each biological replicate (n=5 per hMSC tissue source) used to assemble cumulative vi-SNE maps (see figure 5B).

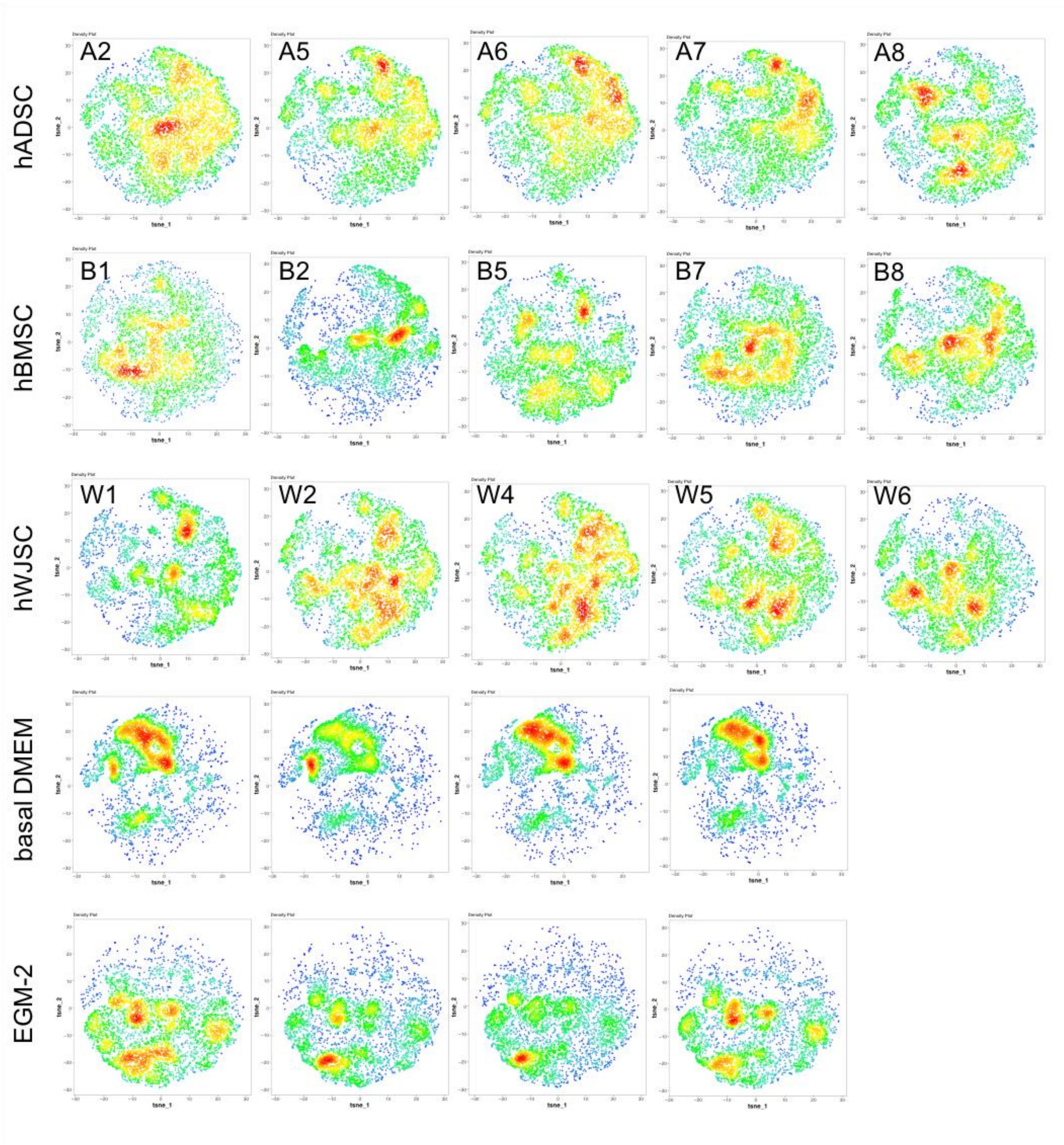

Supplementary figure S12: Density maps from HUVEC after hMSC CM co-incubation. Density maps from each biological replicate (n=5 per hMSC tissue source) used to assemble cumulative density maps (see figure 5B).

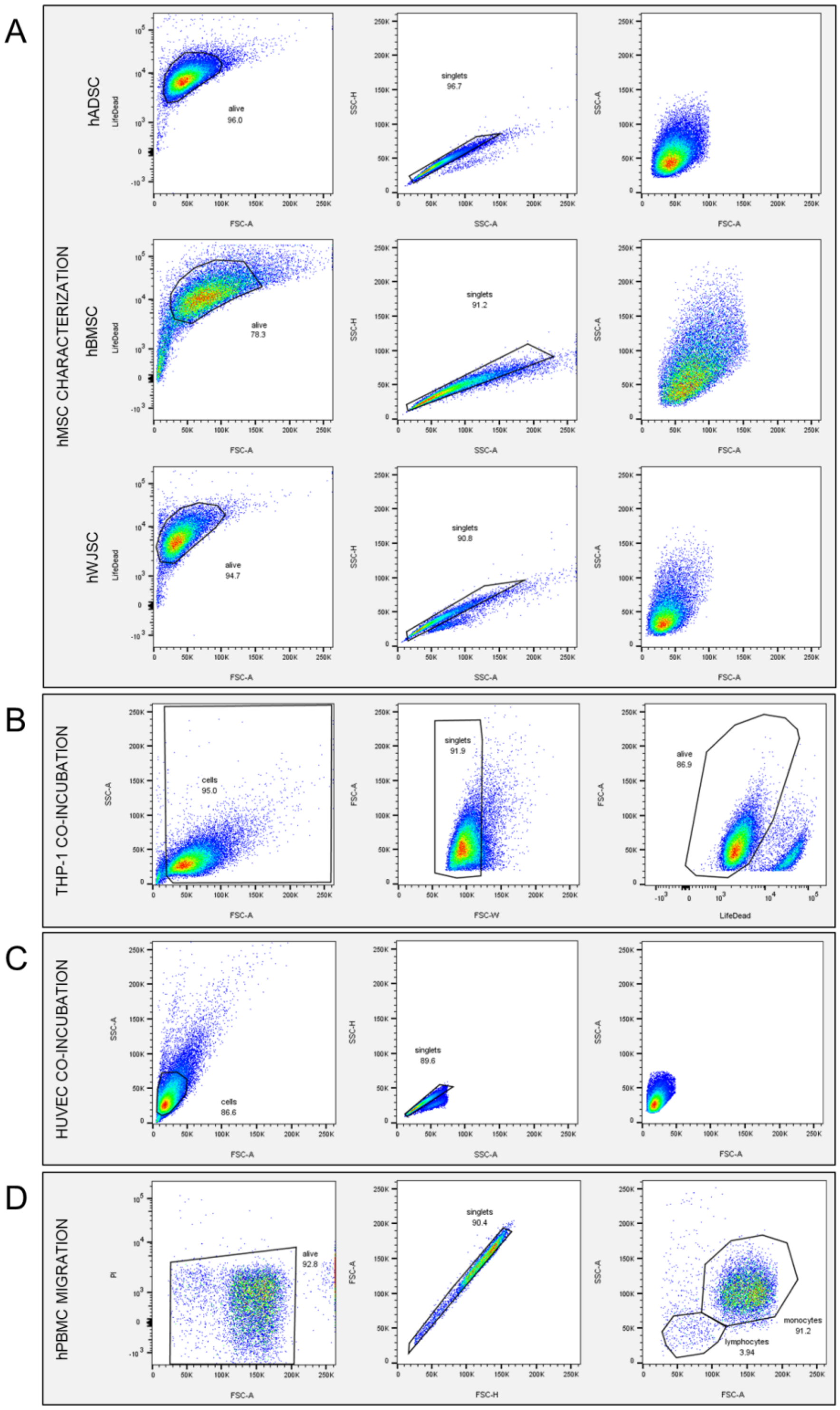

Supplementary figure S13: Flow cytometry gating strategies. Gating strategy for viable and single cells before generating bidimensional t-SNE maps and applying PhenoGraph algorithm to analyze A) characteristic hMSCs surface markers (see figure 1, supplementary figure S2), B) thp-1 phenotype after hMSC CM co-incubation (see figure 5, supplementary figure S6-S8), and C) HUVEC phenotype after hMSC CM co-incubation (see figure 6, supplementary figure S10-S12). D) Gating strategy to define the migration and phenotype of hPBMCs towards hMSC CM (figure 5, supplementary figure S5).

Antibody	Fluorochrome	Species	Clone	Dilution	Company	Reference No.
CD11b	AlexaFluor594	rat anti-human	clone M1/70	1:166	Biolegend, USA	101254
CD11c	PerCP-Cy5	mouse-anti human	clone 3.9	1:100	Biolegend, USA	301609
CD14	FITC	mouse anti-human	clone MϕP	1:6	BD Bioscience, USA	345784
CD14	PerCP	mouse anti-human	clone HCD14	1:100	Biolegend, USA	325631
CD16	PerCP-Cy5.5	mouse anti-human	clone 3G8	1:20	BD Bioscience, USA	560717
CD16	AlexaFluor700	mouse anti-human	clone B73.1	1:166	Biolegend, USA	360717
CD31	V450	mouse-anti human	clone WM59	1:20	BD Bioscience, USA	561653
CD34	PE	mouse anti-human	clone 581	1:2.5	BD Bioscience, USA	555822
CD36	BV605	mouse anti-human	clone CB38	1:166	BD Bioscience, USA	563518
CD45	BV605	mouse anti-human	clone HI30	1:10	BD Bioscience, USA	564047
CD73	Pacific Blue	mouse anti-human	clone AD2	1:20	Biolegend, USA	344012
CD90	PerCP-Cy5.5	mouse anti-human	clone 5E10	1:10	BD Bioscience, USA	561557
CD105	APC	mouse anti-human	clone 266	1:10	BD Bioscience, USA	562408
CD105	FITC	mouse-anti human	clone 43A3	1:20	Biolegend, USA	323204
CD144	PerCP-Cy5.5	mouse-anti human	clone 55-7H1	1:20	BD Bioscience, USA	561566
CD202b	PE	mouse anti-human	clone 33.1	1:20	Biolegend, USA	334206
SRA-I	PE	mouse-anti human	clone REA148	1:166	Miltenyi Biotec, USA	EA460
Viability	Propidium Iodide			1:1000	Abcam, UK	ab14083
Viability	Zombie Aqua			1:1000	Biolegend, USA	423101

Supplementary table S2: Antibody list
